# Supplementary material for: Pharmacogenetic Dose Modeling Based on CYP2C19 Allelic Phenotypes
Source: Pharmaceutics. 2022 Dec 16;14(12):2833. doi: 10.3390/pharmaceutics14122833 (PMC9781550; doi:10.3390/pharmaceutics14122833)
Supplement: Supplementary file 1 [file pharmaceutics-14-02833-s001.zip › SupplFile S2.pdf]

Activity scores

Pharmacogenetic dose modelling, the authors

2022-11-22

This markdown contains the script to produce **Supplementary File S2**.

Preliminary exploration of data

In the figure below, estimated dose adjustments were plotted as a function of phenotype, coded in expression grades of equal intervals from the PM to the UM phenotypes.

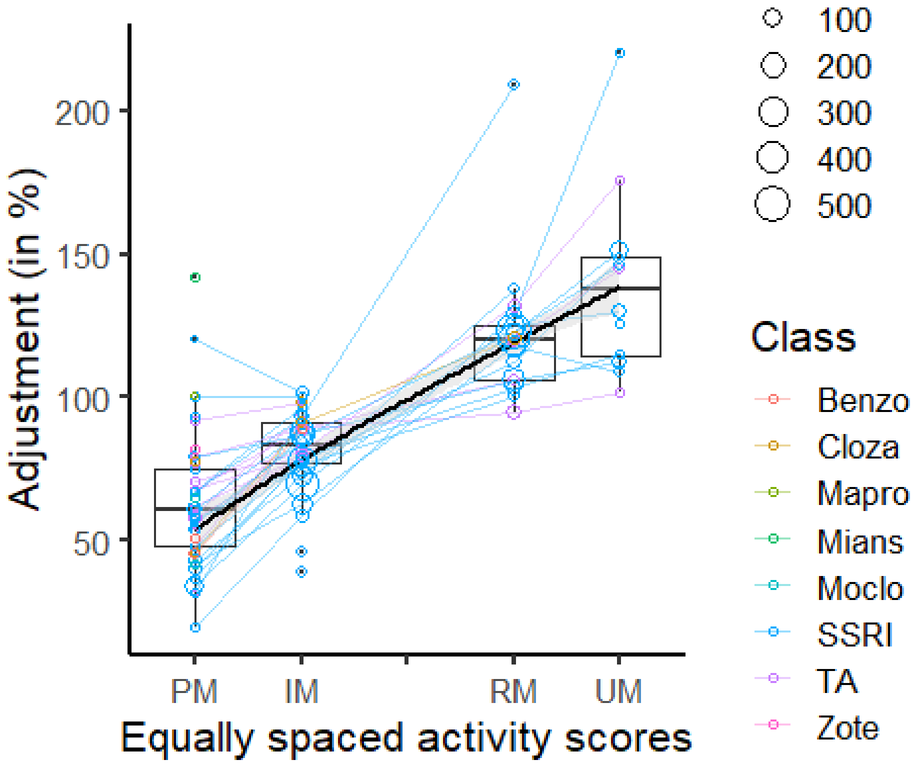

Estimated dose adjustments, equally spaced phenotypic groups

Several properties of the data are apparent from this plot. First, some substances appear not to be metabolized by CYP2C19. One study on mianserine (by Dahl et al. 1994) provides an adjustment that strongly contradicts physiological expectations, increasing dose by about 45% in PMs instead of decreasing. Second, the fitted line is a cubic polynomial, and shows the effect to be almost linear. Third, the spread upwards of the observations seems to be too large.

In the following, we address these problems in a series of steps. First, we will estimate mean dose adjustments in each allelic phenotype separately to compute activity scores from the data. In this computation, we excluded data from substances that appear not to be

metabolized by CYP2C19 (mianserine, maprotiline, fluoxetine and fluvoxamine). Second, we will test the linearity of the effect of these activity scores on dose adjustments by adding quadratic and cubic polynomials. Finally, we will evaluate the need of a logarithmic transformation of the data.

Activity scores

We first looked at the activity scores as may be estimated from these data by fitting a model where phenotypes are qualitative levels of a single factor, using studies as a random effect (to adjust for possible systematic differences in adjustment levels). This amounts to estimating the mean adjustements in each allelic group after allowing for random effects and possible other confounders.

To estimate activity scores, we fix the EM group to zero, and set the effect of the IM group to -1 (as this group has one *2 allele). Note that the base and scale of a measure scale must be chosen by convention. We based the scale to the IM group because there are more data in this group than in the PM and RM groups. To weight individual studies appropriately, we estimate variance components of the variability within and between studies,

$$adjustment_i = phenotype_i + study_{j[i]} + \epsilon_i, i = 1, \ldots, N, j = 1, \ldots, M$$

$$study_j \sim N(0, \sigma_{studies}),$$

where i indexes the N datapoints and j the M studies.

In this and all subsequent models, we face the problem of weighting datapoints by the amount of information provided by the sample size of the original studies. Since only information on sample size is provided in most studies, we assumed equal within-datapoints variance and modelled the variance of the datapoints as normally distributed with two variance components,

$$\epsilon_i \sim N(0, \sigma_w^2/n_i + \sigma_b^2), i = 1, \ldots, N,$$

where ϵ_i is the residual error, n_i is the known number of observations in the datapoint in the ith sample, and σ_w^2 and σ_b^2 are the within-datapoint and between-datapoint variances to be estimated from the data (this model is specified in levels.stan and levels_cov.stan).

```
#get rid of EM measurements and keep only putative substrates
cypx <- filter(cyp, Phenotype != "EM" & Selection == TRUE & Status == "OK")

#identify substances for which we have estimates for all allelic phenotypes
substances <- cypx %>% select(StudyID, Substance, Phenotype) %>%
  group_by(Substance, Phenotype) %>%
  summarize(count = n_distinct(Phenotype)) %>%
  summarize(countphens = sum(count)) %>% filter(countphens > 3)

## `summarise()` has grouped output by 'Substance'. You can override using
the
## `.groups` argument.
```

```
#restrict the analysis to these substances
cypx <- semi_join(cypx, substances, by = "Substance")

#create a dummy variable for levels of phenotype as factor
phen <- cypx$StudyID
phen[cypx$Phenotype == "IM"] <- 1
phen[cypx$Phenotype == "PM"] <- 2
phen[cypx$Phenotype == "RM"] <- 3
phen[cypx$Phenotype == "UM"] <- 4

#create indicator variable for studies included in dataset
studyID <- cypx$StudyID
counter = 1
for (i in 2:nrow(cypx)) {
  if (cypx$StudyID[i-1] != cypx$StudyID[i])
    counter <- counter + 1
  studyID[i] <- counter
}

#set up variables for stan
dat <- list(
  N = nrow(cypx),
  Q = 4,
  M = max(studyID),

  Adjustment = cypx$Adjustment,
  Phenotype = phen,
  Study = studyID,
  nobs = cypx$Size
)
dat$logsigmaloc <- 4
dat$logsigmascale <- 0.25
bayesfit <- stan("levels.stan", data = dat, seed = 142, iter = 2000)

# display estimates of activity scores computed relative to IM
cf = select(as.data.frame(bayesfit), matches("phenotypes"))
ActScoreIM <- cf[,1] * -1
ActScore <- list()
ActScore$EM <- 0
ActScore$IM <- -1
ActScore$PM <- cf[,2] / ActScoreIM
ActScore$RM <- cf[,3] / ActScoreIM
ActScore$UM <- cf[,4] / ActScoreIM
ActScore$star17 <- (ActScore$RM * 2 + ActScore$UM) / 4
ActScore <- as.data.frame(ActScore)
Scores <- sapply(ActScore, median, simplify = "array")
Scores <- rbind(Scores, sapply(ActScore, quantile,
                               probs = 0.05, simplify = "array"))
Scores <- rbind(Scores, sapply(ActScore, quantile,
                               probs = 0.95, simplify = "array"))
```

```
row.names(Scores) <- c("median", "5% lower", "95% upper")
print(Scores, digits = 3)

##           EM IM    PM    RM   UM star17
## median     0 -1 -1.96 0.989 1.76  0.933
## 5% lower   0 -1 -3.13 0.520 1.09  0.601
## 95% upper  0 -1 -1.36 1.806 2.93  1.561

rm(ActScoreIM)
rm(ActScore)
```

This analysis shows that, by setting EM to zero and IM (genotype *1/*null) to -1, PM (genotype *null/*null) has an approximate score of -2, RM (genotype *1/*17) of 1, and UM (genotype *17/*17) of 1.8. The credibility intervals are wide, however; these results are broadly consistent with current assumptions on CYP2C19 activity scores.

Methods to define allelic phenotypes

Studies varied in the way they defined the phenotypic groups. Old studies used phenotyping substrates and only discriminated between PM and EM. The PM determined by phenotyping corresponds to the a genotype consisting of homozygous alleles with zero CYP2C19 enzyme activity (*null/*null). The IM group consists of one active and one inactive allele (*1/*null) of CYP2C19, but in the IM group, the majority of the (older) studies (n=28) pooled data from individuals that might have been carriers of the *17 allele, because the studies performed before the identification of CYP2C19*17 in the year 2006 did not discriminate *17/*null from *1/*null genotypes. One study pooled the PM genotype (homozygous *2 carriers) into the IM group (Islam et al. 2022). The RM group is defined as genotype *1/*17, some studies did not discriminate between the UM and RM groups and pooled possible carriers of the homozygous *17 genotype into one group. The table below shows the number of such studies for substrates where we have data for all phenotypic groups.

```
phgroups <- select(cypx, matches("^(I|R).*\\d$")) %>% sapply(sum) %>% print()

##   IM_17   IM_22 RM_1717   RM_11
##      17       1       3       2
```

As one can see, the estimation of the IM phenotype might be affected by pooling *17 homozygous carriers in the IM group. The number of studies with pooling from other groups may be too small to be able to estimate the effects of pooling. It turns out that it is possible to fit models only to estimate effects of defining the IM and RM phenotypes while pooling *17 homozygous carriers in these groups.

We therefore repeated the analysis with these two confounders.

```
covs <- as.matrix(select(cypx, IM_17, RM_1717))
dat$Covariates <- covs
dat$K <- ncol(covs)
bayesfit <- stan("levels_covs.stan", data = dat, seed = 142, iter = 2000)
print(bayesfit, par = "covcoefs")
```

```
## Inference for Stan model: anon_model.
## 4 chains, each with iter=2000; warmup=1000; thin=1;
## post-warmup draws per chain=1000, total post-warmup draws=4000.
##
##                mean se_mean    sd   2.5%   25%   50%   75% 97.5% n_eff Rhat
## covcoefs[1]  1.68    0.12  8.61 -15.48 -4.16  1.78  7.58 17.97  5025    1
## covcoefs[2] 38.57    0.17 14.05  10.89 29.11 38.57 48.01 65.66  6881    1
##
## Samples were drawn using NUTS(diag_e) at Tue Nov 22 05:29:43 2022.
## For each parameter, n_eff is a crude measure of effective sample size,
## and Rhat is the potential scale reduction factor on split chains (at
## convergence, Rhat=1).
```

```
# display estimates of activity scores computed relative to IM
cf = select(as.data.frame(bayesfit), matches("phenotypes"))
ActScoreIM <- cf[,1] * -1
ActScore <- list()
ActScore$EM <- 0
ActScore$IM <- -1
ActScore$PM <- cf[,2] / ActScoreIM
ActScore$RM <- cf[,3] / ActScoreIM
ActScore$UM <- cf[,4] / ActScoreIM
ActScore$star17 <- (ActScore$RM * 2 + ActScore$UM) / 4
ActScore <- as.data.frame(ActScore)
Scores <- sapply(ActScore, median, simplify = "array")
Scores <- rbind(Scores, sapply(ActScore, quantile,
                               probs = 0.05, simplify = "array"))
Scores <- rbind(Scores, sapply(ActScore, quantile,
                               probs = 0.95, simplify = "array"))
row.names(Scores) <- c("median", "5% lower", "95% upper")
print(Scores, digits = 3)
```

```
##           EM IM    PM    RM    UM star17
## median     0 -1 -1.88 0.663 1.677  0.748
## 5% lower   0 -1 -3.78 0.208 0.966  0.401
## 95% upper  0 -1 -1.19 1.608 3.516  1.612
```

The outcome of this analysis is that the effects of the phenotype definitions are not significant with the exception of the effect of pooling homozygous *17 alleles in to the RM group. As one may expect, studies pooling homozygous *17 carriers into the RM group overestimate its effects; the large value of this overestimation, 38%, is the difference between these studies and those that do not pool (the studies that pool are small and average an adjusted dose of 156% while those that do not pool give 115%). However, the credibility intervals of this effect are enormous.

We therefore opted to define activity scores after excluding studies that pooled the *17 homozygous carriers into the RM group, and verify deviations from linearity by testing a different slope for the *17 carriers in the dataset as a whole. In sum, this gives activity scores of -2, -1, 0.8, and 1.6 for the PM, IM, RM, and UM phenotypic groups.

```r
#create a dummy variable for levels of phenotype as factor
cypx <- filter(cypx, RM_1717 == 0)
phen <- cypx$StudyID
phen[cypx$Phenotype == "IM"] <- 1
phen[cypx$Phenotype == "PM"] <- 2
phen[cypx$Phenotype == "RM"] <- 3
phen[cypx$Phenotype == "UM"] <- 4

#create indicator variable for studies included in dataset
studyID <- cypx$StudyID
counter = 1
for (i in 2:nrow(cypx)) {
  if (cypx$StudyID[i-1] != cypx$StudyID[i])
    counter <- counter + 1
  studyID[i] <- counter
}

#set up variables for stan
dat <- list(
  N = nrow(cypx),
  Q = 4,
  M = max(studyID),

  Adjustment = cypx$Adjustment,
  Phenotype = phen,
  Study = studyID,
  nobs = cypx$Size
)
dat$logsigmaloc <- 4
dat$logsigmascale <- 0.25
bayesfit <- stan("levels.stan", data = dat, seed = 142, iter = 2000)

# display estimates of activity scores computed relative to IM
cf = select(as.data.frame(bayesfit), matches("phenotypes"))
ActScoreIM <- cf[,1] * -1
ActScore <- list()
ActScore$EM <- 0
ActScore$IM <- -1
ActScore$PM <- cf[,2] / ActScoreIM
ActScore$RM <- cf[,3] / ActScoreIM
ActScore$UM <- cf[,4] / ActScoreIM
ActScore$star17 <- (ActScore$RM * 2 + ActScore$UM) / 4
ActScore <- as.data.frame(ActScore)
Scores <- sapply(ActScore, median, simplify = "array")
Scores <- rbind(Scores, sapply(ActScore, quantile,
                               probs = 0.05, simplify = "array"))
Scores <- rbind(Scores, sapply(ActScore, quantile,
                               probs = 0.95, simplify = "array"))
row.names(Scores) <- c("median", "5% lower", "95% upper")
print(Scores, digits = 3)
```

```
##             EM IM     PM     RM    UM star17
## median       0 -1 -1.96 0.699 1.75  0.787
## 5% lower     0 -1 -2.91 0.262 1.13  0.479
## 95% upper    0 -1 -1.42 1.277 2.76  1.264

rm(ActScoreIM)
rm(ActScore)
```

Formally testing linearity

To verify linearity of activity scores we turned to a model of adjustment as a function of activity scores. We followed the following strategy:

- we omitted the constant term, so that the label-relative EM dose is adjusted to itself (zero change)
- we added random effects of activity for substances, but no random effect for the intercept, to constrain the effect of activity scores to pass through zero at the EM group;
- we coded the activity scores as -2 (PM), -1 (IM), 0.8 (RM), and 1.6 (UM) following the results of the previous section.

The basic model is

$$adjustment_i = activity\_score + study_{j[i]} + \epsilon_i, i = 1, \ldots, N, j = 1, \ldots, M$$

$$study \sim N(0, \sigma_{studies})$$

The residual variance of the ϵ_i is modelled as before (this model is specified in lmact_covs.stan).

Test for different scores *17 and *null

Here, we test that the slope of the RM and UM phenotypes differs from the slope of the IM and PM phenotypes with the activity scores given above.

```
#get rid of EM measurements and keep only putative substrates
cypsel <- filter(cyp, Phenotype != "EM" & Selection == TRUE & Status == "OK")

#define a different slope for *17 carriers
covs <- as.matrix(cypsel %>%
    transmute(star17 = Activity * (Phenotype == "RM" | Phenotype == "UM")))

#create indicator variable for studies included in dataset
studyID <- cypsel$StudyID
counter = 1
for (i in 2:nrow(cypsel)) {
  if (cypsel$StudyID[i-1] != cypsel$StudyID[i])
    counter <- counter + 1
  studyID[i] <- counter
}

#set up variables for stan
```

```
dat <- list(
  N = nrow(cypsel),
  K = ncol(covs),
  M = max(studyID),

  Adjustment = cypsel$Adjustment,
  Activity = cypsel$Activity,
  Covariates = covs,
  Study = studyID,
  nobs = cypsel$Size
)
dat$logsigmaloc <- 4
dat$logsigmascale <- 0.25
star17fit <- stan("lmact_covs.stan", data = dat, seed = 142, iter = 2000)
print(star17fit, par = "covcoefs")

## Inference for Stan model: anon_model.
## 4 chains, each with iter=2000; warmup=1000; thin=1;
## post-warmup draws per chain=1000, total post-warmup draws=4000.
##
##              mean se_mean  sd  2.5%  25%  50%  75% 97.5% n_eff Rhat
## covcoefs[1] 5.04    0.06 4.1 -3.04 2.28 5.08 7.79 13.03  4378    1
##
## Samples were drawn using NUTS(diag_e) at Tue Nov 22 05:30:28 2022.
## For each parameter, n_eff is a crude measure of effective sample size,
## and Rhat is the potential scale reduction factor on split chains (at
## convergence, Rhat=1).
```

The minor difference in the slope of activity (given by covcoefs[1]) is now too small to be detectable in this model.

Nonlinearity

We now evaluate these scores by fitting a model with quadratic and cubic terms, to test for deviations from non-linearity. First, the model with cubic effects:

```
#get rid of EM measurements and keep only putative substrates
cypsel <- filter(cyp, Phenotype != "EM" & Selection == TRUE & Status == "OK")

#define quandratic and cubic effects, and add homozygous *17 in RM as
confounder
covs <- as.matrix(cypsel %>% transmute(quadratic = Activity^2,
cubic=Activity^3,
                                       RM_1717 = RM_1717))

#create indicator variable for studies included in dataset
studyID <- cypsel$StudyID
counter = 1
for (i in 2:nrow(cypsel)) {
  if (cypsel$StudyID[i-1] != cypsel$StudyID[i])
    counter <- counter + 1
  studyID[i] <- counter
```

```
}

#set up variables for stan
dat <- list(
  N = nrow(cypsel),
  K = ncol(covs),
  M = max(studyID),

  Adjustment = cypsel$Adjustment,
  Activity = cypsel$Activity,
  Covariates = covs,
  Study = studyID,
  nobs = cypsel$Size
)
dat$logsigmaloc <- 4
dat$logsigmascale <- 0.25
nonlin3 <- stan("lmact_covs.stan", data = dat, seed = 142, iter = 2000)
print(nonlin3, par = "covcoefs")

## Inference for Stan model: anon_model.
## 4 chains, each with iter=2000; warmup=1000; thin=1;
## post-warmup draws per chain=1000, total post-warmup draws=4000.
##
##              mean se_mean    sd  2.5%   25%   50%   75% 97.5% n_eff Rhat
## covcoefs[1]  1.50    0.03  1.47 -1.34  0.48  1.49  2.51  4.33  3405    1
## covcoefs[2]  1.40    0.03  1.41 -1.32  0.46  1.38  2.33  4.26  2420    1
## covcoefs[3] 27.65    0.16 10.84  6.19 20.48 27.70 34.83 49.28  4685    1
##
## Samples were drawn using NUTS(diag_e) at Tue Nov 22 05:30:53 2022.
## For each parameter, n_eff is a crude measure of effective sample size,
## and Rhat is the potential scale reduction factor on split chains (at
## convergence, Rhat=1).
```

The cubic effect is covcoefs[2] (in this analysis, we also adjust for pooling homozygous *17 in the RM group, which gives covcoefs[3]). Now the quadratic effects:

```
#define quandratic and cubic effects, and add homozygous *17 in RM as
confounder
covs <- as.matrix(cypsel %>%
                transmute(quadratic = Activity^2, RM_1717 = RM_1717))

#create indicator variable for studies included in dataset
studyID <- cypsel$StudyID
counter = 1
for (i in 2:nrow(cypsel)) {
  if (cypsel$StudyID[i-1] != cypsel$StudyID[i])
    counter <- counter + 1
  studyID[i] <- counter
}

#set up variables for stan
```

```r
dat <- list(
  N = nrow(cypsel),
  K = ncol(covs),
  M = max(studyID),

  Adjustment = cypsel$Adjustment,
  Activity = cypsel$Activity,
  Covariates = covs,
  Study = studyID,
  nobs = cypsel$Size
)
dat$logsigmaloc <- 4
dat$logsigmascale <- 0.25
nonlin2 <- stan("lmact_covs.stan", data = dat, seed = 142, iter = 2000)
print(nonlin2, par = "covcoefs")
```

```
## Inference for Stan model: anon_model.
## 4 chains, each with iter=2000; warmup=1000; thin=1;
## post-warmup draws per chain=1000, total post-warmup draws=4000.
##
##              mean se_mean    sd  2.5%   25%   50%   75% 97.5% n_eff Rhat
## covcoefs[1]  0.61    0.02  1.18 -1.77 -0.17  0.61  1.39  2.89  5248    1
## covcoefs[2] 26.08    0.13 10.71  4.98 18.93 26.05 33.38 47.13  7012    1
##
## Samples were drawn using NUTS(diag_e) at Tue Nov 22 05:31:18 2022.
## For each parameter, n_eff is a crude measure of effective sample size,
## and Rhat is the potential scale reduction factor on split chains (at
## convergence, Rhat=1).
```

The quadratic effect is covcoefs[1] (covcoefs[2] is again the pooling of the homozygous *17 within the RM group). None of these nonlinear terms are significant. We therefore accept the activity scores we defined above.

Here is the boxplot of the data, this time with the new activity score and the polynomial fit.

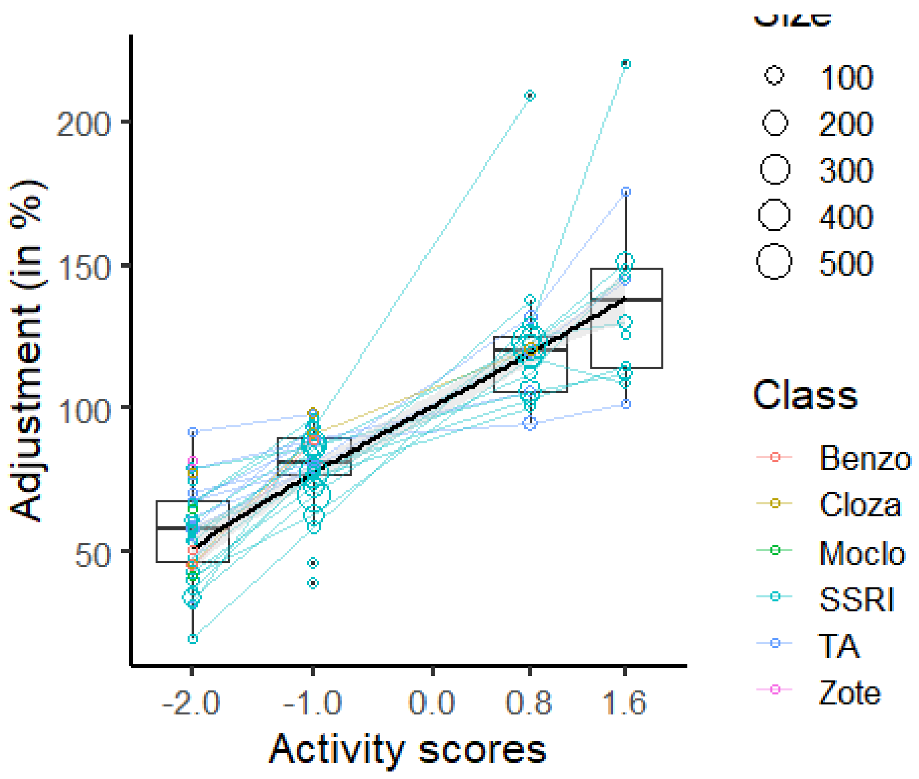

Adjustments phenotypic groups with activity scores

As one can see, the polynomial predictor gives now a linear fit, showing the non-linear terms to be redundant.

Log transformation

When considering a log-transformation of the adjustments, we obtain an effect that is no longer linear for equally spaced activity scores. The plot below reports both the linear (in black) and cubic polynomial fit, showing the latter to deviate from the linear fit.

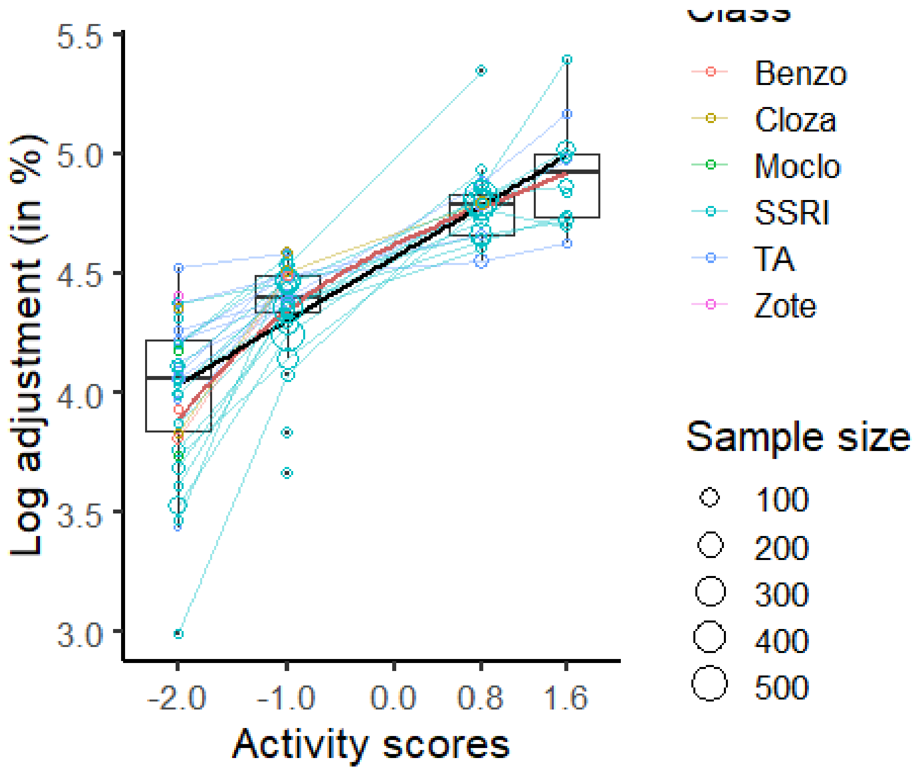

Fitted line: black: original data, color: log-transformed data

This plots shows that the log transform would not be appropriate in this dataset.

Effects of studies

Coding EMs by pooling oher phenotypes

In some studies, the EM allelic group was defined while pooling within it other alleles, the *2 or the *17:

```
phgroups <- filter(studies, Status == "OK") %>%
  select(matches("^EM.*\\d$")) %>% sapply(sum) %>% print()

## EM_17  EM_2
##    32     9
```

This pooling affects all other phenotypic groups, because the EM estimate is the reference point in the original studies, affecting all other phenotypes at once. We therefore investigated the effects of EM pooling on the estimated effects of activity scores (not on the estimates of individual phenotypic groups as in a previous section. In this analysis, we also adjust for pooling homozygous *17 in the RM group).

```
#get rid of EM measurements and keep only putative substrates
cypsel <- filter(cyp, Phenotype != "EM" & Selection == TRUE & Status == "OK")

#EM_2 not estimable, too few data
covs <- as.matrix(select(cypsel, EM_17xAct2, EM_17xAct17, EM_2xAct2,
```

```
RM_1717))

#create indicator variable for studies included in dataset
studyID <- cypsel$StudyID
counter = 1
for (i in 2:nrow(cypsel)) {
  if (cypsel$StudyID[i-1] != cypsel$StudyID[i])
    counter <- counter + 1
  studyID[i] <- counter
}

#set up variables for stan
dat <- list(
  N = nrow(cypsel),
  K = ncol(covs),
  M = max(studyID),

  Adjustment = cypsel$Adjustment,
  Activity = cypsel$Activity,
  Covariates = covs,
  Study = studyID,
  nobs = cypsel$Size
)
dat$logsigmaloc <- 4
dat$logsigmascale <- 0.25
EMfit <- stan("lmact_covs.stan", data = dat, seed = 142, iter = 2000)

print(EMfit, par = c("covcoefs", "sigma_within", "sigma_betw"))

## Inference for Stan model: anon_model.
## 4 chains, each with iter=2000; warmup=1000; thin=1;
## post-warmup draws per chain=1000, total post-warmup draws=4000.
##
##                 mean se_mean    sd   2.5%    25%    50%    75% 97.5% n_eff
Rhat
## covcoefs[1]   -5.26    0.05  3.25 -11.55  -7.45  -5.23  -3.11  1.04  4367
1
## covcoefs[2]  -16.31    0.11  8.47 -33.24 -21.82 -16.15 -10.87  0.96  6265
1
## covcoefs[3]   -0.96    0.06  4.71 -10.19  -4.13  -0.99   2.25  8.41  5857
1
## covcoefs[4]   23.42    0.13 10.34   3.10  16.56  23.26  30.12 44.42  6365
1
## sigma_within  35.25    0.08  5.93  24.71  31.04  34.95  39.05 47.59  5685
1
## sigma_betw    17.05    0.03  1.96  13.52  15.69  16.93  18.30 21.16  5701
1
##
## Samples were drawn using NUTS(diag_e) at Tue Nov 22 05:31:43 2022.
## For each parameter, n_eff is a crude measure of effective sample size,
```

```
## and Rhat is the potential scale reduction factor on split chains (at
## convergence, Rhat=1).
```

Pooling heterozygous *2 in the EM group (which happened in the old phenotyping studies that pooled all genotypes other than *null/*null into one group of EM) appears to have no effect on estimate of activity scores. Instead, the pooling of *17 carriers in the EM group (=*1/*17 or *17/*17), which happened in the older studies that genotyped for the *null alleles but not for *17 alleles, leads to smaller effects of activity scores on adjustments in the UM phenotype. However, these effects fail to reach significance.

Pharmacokinetic parameters

We also tested parameters of the study design that might affect the estimation of the CYP2C19 phenotype from pharmacokinetic data. One parameter consisted of the pharmacokinetic measurement used to estimate the dose adjustments. Some studies (usually performed in healthy participants as pharmacokinetic study) used AUC or Clearance, other studies (usually in patients and at naturalistic conditions) used Css, the dose corrected plasma concentration at steady state. We therefore tested the influence of the pharmacokinetic parameter given in the studies and the participants (healthy versus patients).

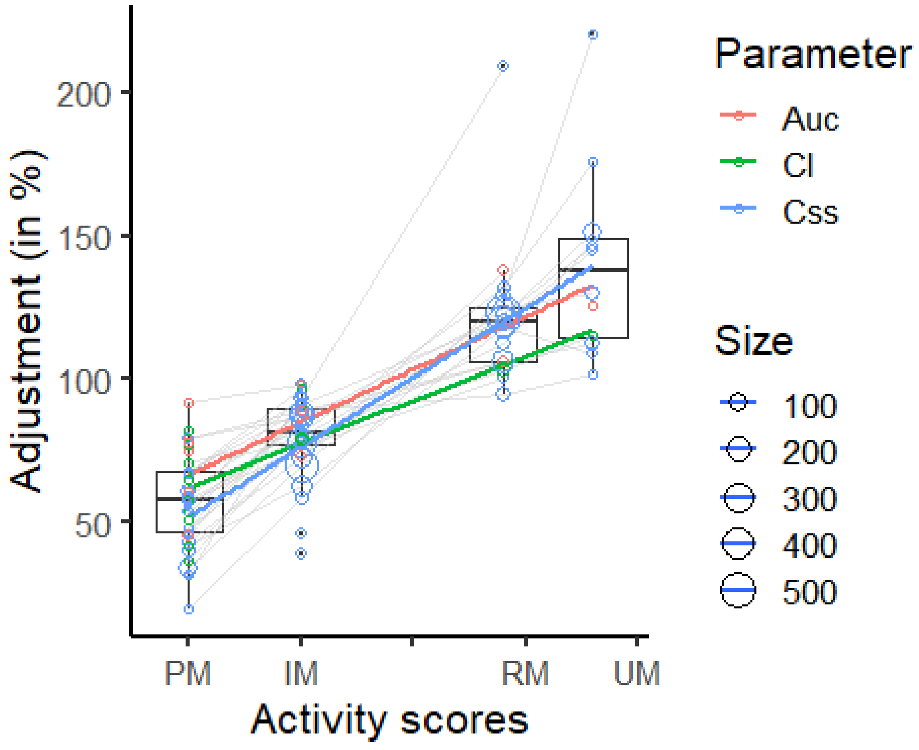

This plot suggests a slight effect of these parameters on the slope. Css appears to overestimate the allelic effect relative to both Auc and Cl.

We repeat the fit with a Bayesian model to weight the observations by the estimated components of the residuals.

```
#get rid of EM measurements and keep only putative substrates
cypsel <- filter(cyp, Phenotype != "EM" & Selection == TRUE & Status == "OK")

#covariates
covs <- as.matrix(cypsel %>%
          mutate(Auc = Activity * (Parameter == "Auc"),
                 Cl = Activity * (Parameter == "Cl")) %>%
            select(RM_1717, Auc, Cl))

#create indicator variable for studies included in dataset
studyID <- cypsel$StudyID
counter = 1
for (i in 2:nrow(cypsel)) {
  if (cypsel$StudyID[i-1] != cypsel$StudyID[i])
    counter <- counter + 1
  studyID[i] <- counter
}

#set up data for stan
dat <- list(
  N = nrow(cypsel),
  K = ncol(covs),
  M = max(studyID),

  Adjustment = cypsel$Adjustment,
  Activity = cypsel$Activity,
  Covariates = covs,
  Study = studyID,
  nobs = cypsel$Size
)
dat$logsigmaloc <- 4
dat$logsigmascale <- 0.25
paramfit <- stan("lmact_covs.stan", data = dat, seed = 142, iter = 2000)
print(paramfit, par = "covcoefs")

## Inference for Stan model: anon_model.
## 4 chains, each with iter=2000; warmup=1000; thin=1;
## post-warmup draws per chain=1000, total post-warmup draws=4000.
##
##                mean se_mean    sd   2.5%    25%   50%   75% 97.5% n_eff Rhat
## covcoefs[1] 26.41    0.14 10.59   5.86  19.33 26.34 33.39 47.46  5493    1
## covcoefs[2] -8.07    0.06  4.46 -16.65 -11.04 -8.04 -5.15  0.89  5669    1
## covcoefs[3] -4.38    0.05  3.74 -11.87  -6.86 -4.40 -1.92  2.93  5945    1
##
## Samples were drawn using NUTS(diag_e) at Tue Nov 22 05:32:09 2022.
## For each parameter, n_eff is a crude measure of effective sample size,
## and Rhat is the potential scale reduction factor on split chains (at
## convergence, Rhat=1).
```

(In the printout, covcoefs[1] is the effect of RM pooling, covcoefs[2] of Auc relative to Css and covcoefs[3] of Cl). The model shows the slope of Css to be larger than in the other methods by 6-8%. This is a relatively small effect, and indeed it fails to reach significance.

We decided against including this parameter in the final model not only because it is small, but also because this estimate may be biased by the confounder given by previous knowledge that the CYP2C19 effect is present and large. This may have been an incentive to conduct large studies in clinical samples.

Single/multiple studies (dosage)

Pharmacokinetic parameters were determined in some studies with single and in some studies with multiple doses. This variable is almost the same as studies conducted on patients or healthy volunteers, which cannot be studies separately.

It would not be expected for single and multiple dose methods to influence the magnitude of the CYP2C19 pathway coefficient, but its precision. Hence, we modified the model and tested a heteroscedastic variance within studies depending on study type, single or multiple dose. The model for the residual errors replaces the variance within σ_w^2 with σ_k^2,

$$\epsilon_i \sim N(0, \sigma_k^2/n_i + \sigma_b^2), k = 1,2$$

where k indexes single and multiple dose, and i indexes the datapoints as before, $i = 1,2, \dots, N$. We keep the adjustment fo RM pooling in the model.

```
#get rid of EM measurements and keep only putative substrates
cypsel <- filter(cyp, Phenotype != "EM" & Selection == TRUE & Status == "OK")

#covariates
covs <- as.matrix(cypsel %>% mutate(Auc = Activity * (Parameter == "Auc"))
%>%
                    select(RM_1717))

#create indicator variable for studies included in dataset
studyID <- cypsel$StudyID
counter = 1
for (i in 2:nrow(cypsel)) {
  if (cypsel$StudyID[i-1] != cypsel$StudyID[i])
    counter <- counter + 1
  studyID[i] <- counter
}

#set up data for stan
dat <- list(
  N = nrow(cypsel),
  K = ncol(covs),
  M = max(studyID),

  Adjustment = cypsel$Adjustment,
  Activity = cypsel$Activity,
  Covariates = covs,
```

```
  Study = studyID,
  nobs = cypsel$Size,
  sigmaidx = as.integer(cypsel$Dosage == "SD") + 1
)
dat$logsigmaloc <- 4
dat$logsigmascale <- 0.25
sdfit <- stan("lmact_covs_wghtex.stan", data = dat, seed = 142, iter = 2000)
print(sdfit, par = c("covcoefs", "sigma_within", "sigma_betw"))
```

```
## Inference for Stan model: anon_model.
## 4 chains, each with iter=2000; warmup=1000; thin=1;
## post-warmup draws per chain=1000, total post-warmup draws=4000.
##
##                  mean se_mean    sd  2.5%   25%   50%   75% 97.5% n_eff
Rhat
## covcoefs[1]     25.11    0.13 10.37  5.14 17.92 25.19 32.10 45.11  6787
1
## sigma_within[1] 44.30    0.11  8.22 29.29 38.39 44.02 49.71 61.28  5539
1
## sigma_within[2] 36.86    0.09  7.38 24.43 31.63 36.22 41.25 52.89  6118
1
## sigma_betw      16.79    0.03  2.04 12.97 15.37 16.76 18.14 20.89  5022
1
##
## Samples were drawn using NUTS(diag_e) at Tue Nov 22 05:35:20 2022.
## For each parameter, n_eff is a crude measure of effective sample size,
## and Rhat is the potential scale reduction factor on split chains (at
## convergence, Rhat=1).
```

As expected, there was smaller variability in the studies with single dose (sigma_within[2]) than in those with multiple dose (sigma_within[1]). However, the credibility intervals largely overlapped. Importantly, studies with single dose are more expensive; only small studies are available with single dose.

It is worth noting that there was an association between single/multiple dose and the the pharmacokinetic method:

```
##
##       Auc Cl Css
##    MD   1  6  71
##    SD  13 12   0
```

Hence, the heteroscedastic model of this section also considers modelling effects of parameters on variance rather than mean activity scores.

Conclusion

In conclusion, we should add in the model the effects of pooling both *17 alleles in the RM group. The effects of *17 carriers in the EM or IM groups and of AUC/single or multiple studies are so small they may be neglected. As shown in the figures below, residuals are larger in the PM and UM group, but this may be expected given that the sample sizes are

smaller (this aspect is already taken care of by the model of the residuals). Note also the symmetry of the residuals, confirming that a log transformation of adjustments is not necessary. The random effect of studies shows here reasonable homogeneity.

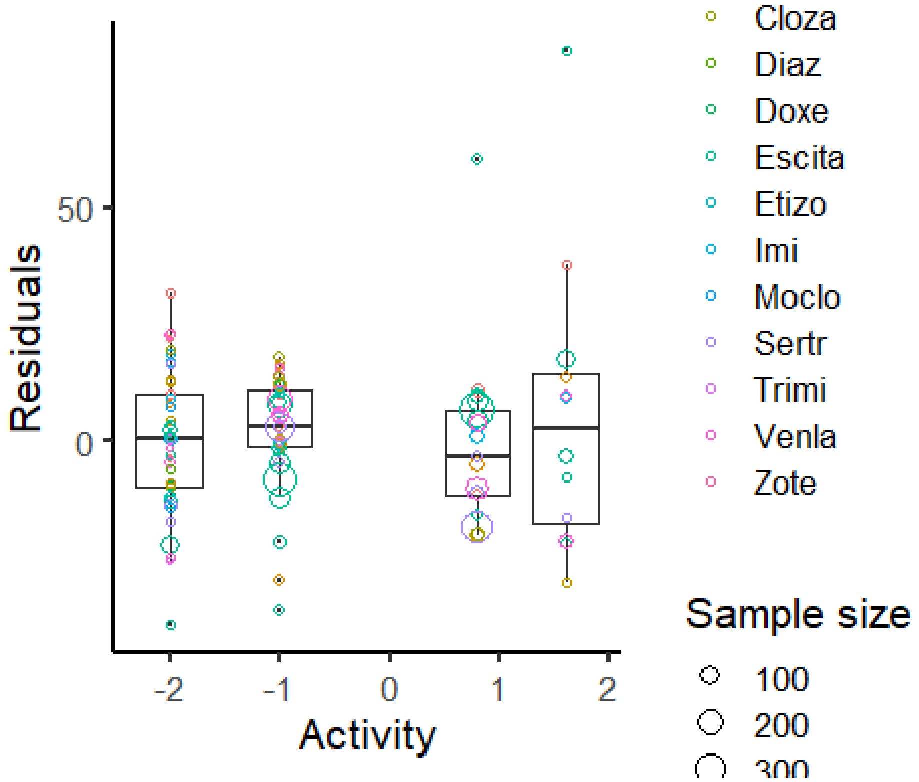


```
hist(as.data.frame(sdfit) %>% select(matches("^studies")) %>%
       map_df(median) %>% as.matrix(),
     xlab = "studies", main = "Random effect of studies",
     breaks = 8)
```

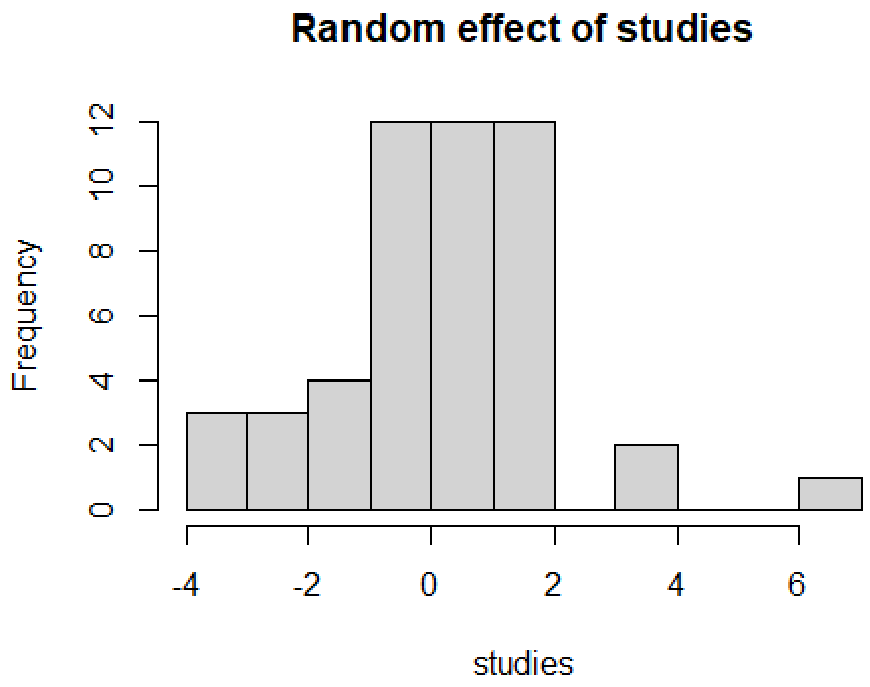

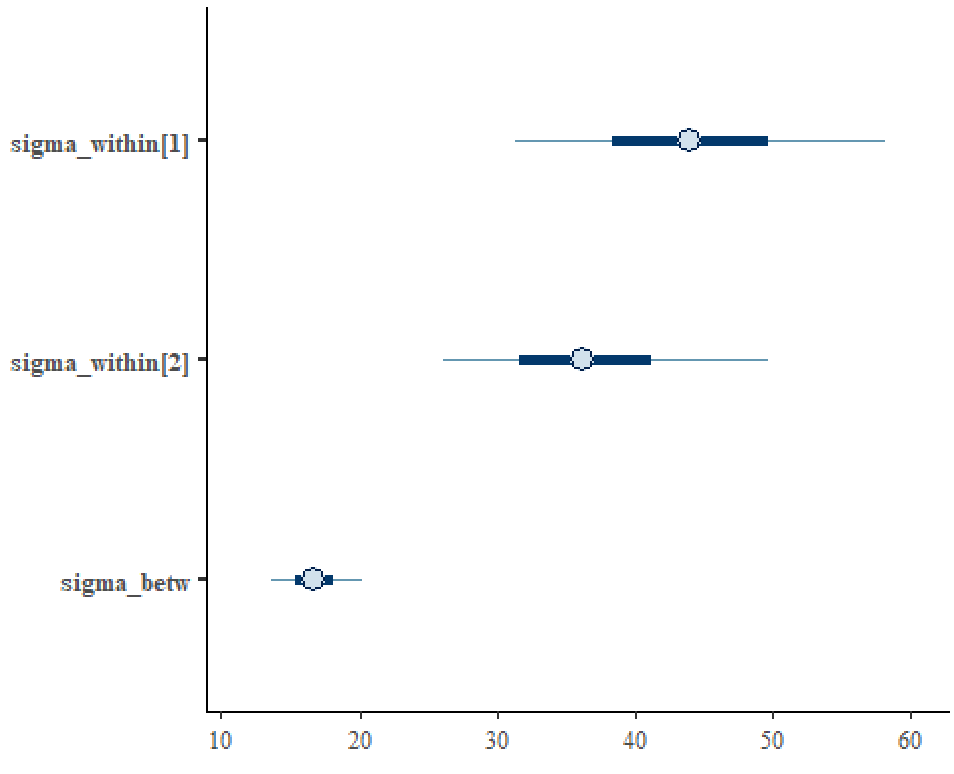

estimates and confidence intervals, residual dispersion

```
disp <- as.data.frame(sdfit) %>% select(matches("dispersion")) %>%
map_dbl(median) %>% as.matrix()
```

```
plot(disp + runif(nrow(cypsel)) ~ log(cypsel$Size), pch = "o", col =
ifelse(cypsel$Dosage == "SD", "blue", "red"))
```

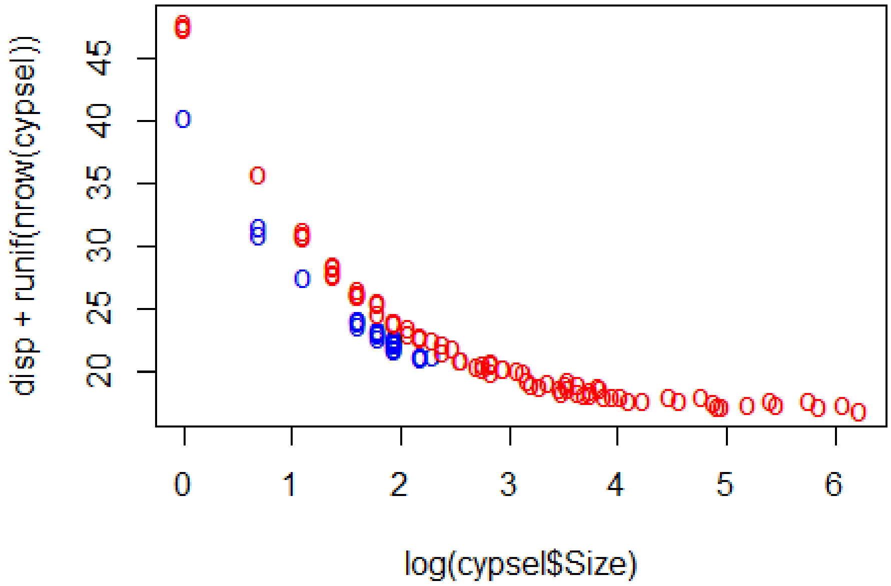

In blue, estimated within datapoint standard deviation of single dose studies, in red, the multiple dose studies, plotted as a function of datapoint sample size. Jitter was added to identify samples.
