# Supplementary material for: Pharmacogenetic Dose Modeling Based on CYP2C19 Allelic Phenotypes
Source: Pharmaceutics. 2022 Dec 16;14(12):2833. doi: 10.3390/pharmaceutics14122833 (PMC9781550; doi:10.3390/pharmaceutics14122833)
Supplement: Supplementary file 1 [file pharmaceutics-14-02833-s001.zip › SupplFile S3.pdf]

Shrinkage models (Gaussian and horseshoe)

Pharmacogenetic dose modelling, the authors

2022-11-22

This markdown contains the script to produce the **Supplementary File S3**.

Random effects substances

We explore here the use of shrinkage to model adjustments for the allelic phenotypes, starting with a standard random effect. The random effects models the interaction between substances and activity scores. This assumes that variation ensuing from differences in CYP2C19 affinity for the substrate, from the importance of the metabolic pathway, and from other unmeasured sources contributed to variation in adjustment levels distributed across a population of substances, from which the substances in the study are a sample.

The assumption is that the dose adjustments for the CYP2C19 phenotype group in a given substance depends on a coefficient expressing the linear increase of the adjustment for a given CYP2C19 activity score. The slope of the activity score effect predicting dose adjustments may be determined based on the fit of a linear model for the phenotype groups that have been measured. Based on this fit, we are able to extrapolate dose adjustments also for the phenotype groups where we had no measurements.

We model the interactions between substances and activity, without including a mean effect of activity scores. As a result, shrinkage takes place in the direction of null effects.

In all these models, data about the EMs have been removed because thy do not provide independent observations (they are used in the computation of the adjustment for the other groups). We ignored the size of the EMs when computing the weighting of the residuals, as there are usually many observations in this group. We omitted the intercept from the model to constrain the fitted adjustment to zero for the EM group.

We do not model studies as a random effect as we do not have an intercept. We should model the slope in this random effect, but there are many substances where only one study was carried out. For this reason, the random effect of studies in slopes is not identifiable relative to the random effect of substances.

In summary, to model adjustment as a function of phenotype, we followed the following strategy:

- we omitted the constant term to constrain the coefficient for EMs to zeros, reflecting the fact that all modeled adjustments were computed by comparing pharmacokinetic data to those of the EM group;
- we added random effects for the interaction activity scores x substances, but no random effect for the intercept;
- we coded the activity scores as -2 (PM), -1 (IM), 0 (EM), 0.8 (RM), and 1.6 (UM):

$$adjustment_i = activity\_score_i : substance_{j[i]} + confounder_i + \epsilon_i,$$

$$activity\_score_i : substance_{j[i]} \sim N(0, \tau^2)$$

with residuals modelled as in the activity scores models as a mixture of two components:

$$\epsilon_i \sim N(0, \sigma_w^2/n_i + \sigma_b^2)$$

where ϵ_i is the residual error, n_i is the known number of observations in the datapoint in the ith sample, and σ_w^2 and σ_b^2 are the within-datapoint and between-datapoint variances to be estimated from the data, and $i = 1, \ldots, N, j = 1, \ldots, M$, where i indexes the N datapoints and j the M studies. The confounder is given by the datapoints on the RM group where homozygous *17 carriers where pooled within this group (this model is in the file rnd_noipct_wght.stan).

```
#parameters for dispersion model of datapoints in studies
adddisppars <- function(lst) {
  lst$logsigmaloc <- 4
  lst$logsigmascale <- 0.25
  lst
}
```

Because we do not assume here that all substances in the literature were substrates of CYP2C19, we include in the sample all compounds on which data were published, for example including also compounds such as mianserine where there is clearly no metabolism by CYP2C19.

In all models below, we checked that the stan diagnostics reported no problem in sampling from the posterior. We note when this is not the case.

```
preds <- cypsel %>%
  pivot_wider(names_from = Substance, values_from = Activity,
              values_fill = 0) %>%
  select(Ami:Mians) %>% as.matrix()
covs <- select(cypsel, RM_1717)
mednames <- colnames(cypsel %>%
  pivot_wider(names_from = Substance, values_from = Activity,
              values_fill = 0) %>%
  select(Ami:Mians));

meds_dat <- list(
  N = nrow(preds),
  K = ncol(preds),
  M = ncol(preds) + ncol(covs),
  nobs = cypsel$Size,

  y = cypsel$Adjustment - 100,
  preds = preds,
  covs = covs
)
meds_dat <- adddisppars(meds_dat)
```

```
rndfit <- stan("rnd_noipct_wght.stan", data = meds_dat, seed = 142)
plot_mikado(rndfit)
```

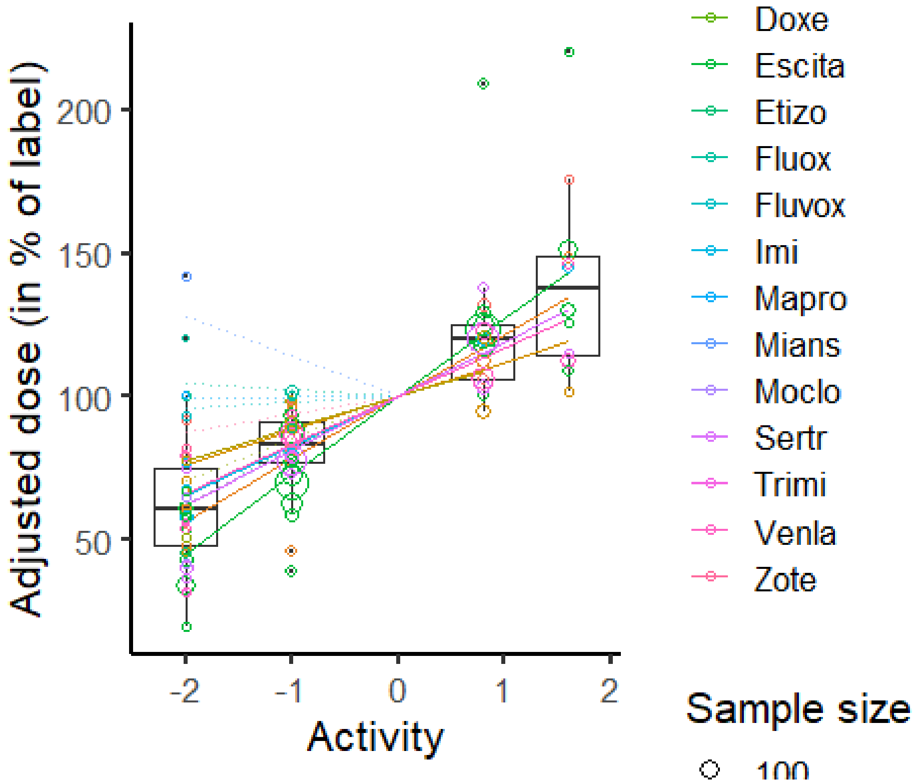


```
plot_intervals(rndfit, "IM")
```

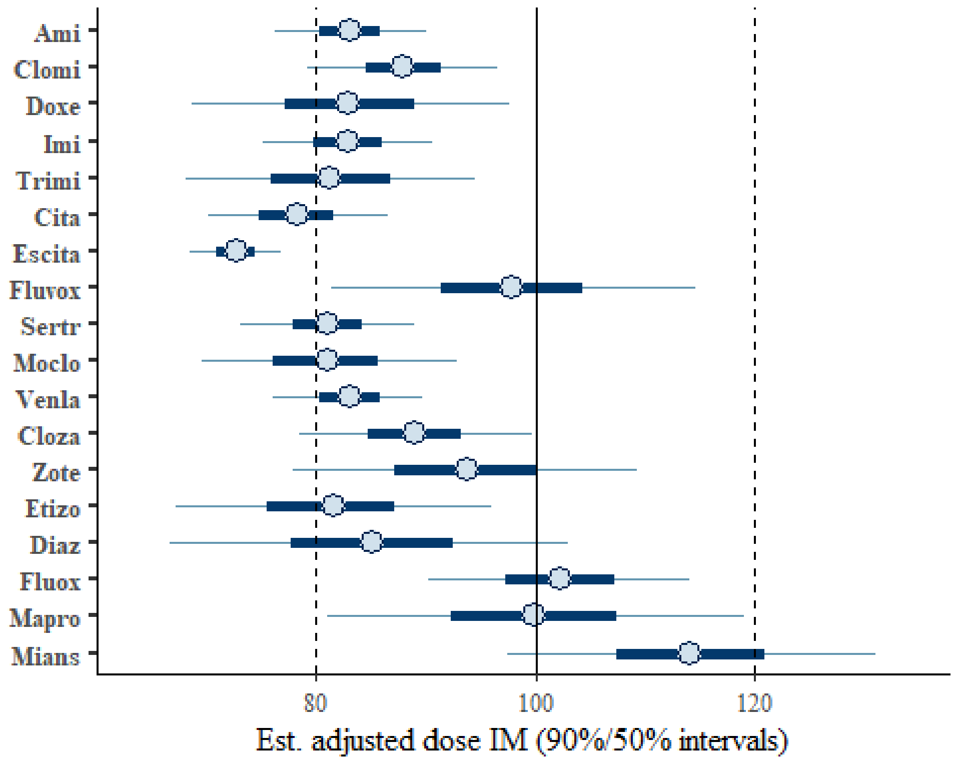

This fit shows some shrinkage towards zero effects, but considerable spread of fitted effects of activity scores for substances. This model picked up several substances as not being substrates of CYP2C19 in that we do not sufficient evidence to exclude a null effect (based on 95% credibility intervals, these compounds include: fluoxetine, fluvoxamine, maprotiline, mianserine, zotepine).

Note that we would have different predicted adjustments if we had modelled the data with a mean activity scores effect, i.e.

$$adjustment_i = mean\_activity\_score + activity\_score_i : substance_{j[i]} + confounder_i + \epsilon_i,$$

In this case, the coefficients of $activity\_score_i : substance_{j[i]}$ encode the *difference* of the strength of CYP2C19 metabolism of $substance_{j[i]}$ from $mean\_activity\_score$. When there is little information on a substance, shrinkage of these difference estimates to zero leads to predicting average activity score effects, not zero effects as in the model adopted in the present study (these average effects are shown in Supplementary File S2 as the fits of activity scores).

Regularized horseshoe

In the regularized horsehoe model, the distribution of the random effect is modelled as a sophisticated ridge (see main paper for details):

$$activity\_score_i : substance_{j[i]} \sim HS\left(0, \tau^2 \xi^2_{j[i]}\right)$$

The rest of the model is the same as in the random effect model above. We set the prior for τ^2 to have approximately 50% of non-zero effects, following the derivation of Piironen & Vehtari (2017) (the model is in the rhs_noicpt_wght.stan file).

```
#Horseshoe settings. We use the resulty by Vehtari et al. to set
#the prior for nonnull coefficients at a 50% rate.
p0 <- 9
p <- 18

addhspars <- function(lst, data) {
  #to set a more pessimistic prior, set
  #global_scale <- 0.001
  lst$scale_global <- p0/(p-p0) / sqrt(nrow(data))
  lst$nu_global  <- 1  #Cauchy for tau

  lst$nu_local   <- 1  #Cauchy for lambdas
  lst$slab_scale <- 2.5
  lst$slab_df    <- 8
  lst
}

#named list for stan. Confounding covariates at the end.
preds <- cypsel %>%
  pivot_wider(names_from = Substance, values_from = Activity,
              values_fill = 0) %>%
  mutate(Activity = get_activity(Phenotype)) %>%
```

```
  dplyr::select(Ami:Mians, RM_1717) %>% as.matrix()
mednames <- colnames(cypsel %>%
  pivot_wider(names_from = Substance, values_from = Activity,
              values_fill = 0) %>%
  dplyr::select(Ami:Mians))

meds_dat <- list(
  N = nrow(preds),
  K = 18,
  M = ncol(preds),
  nobs = cypsel$Size,

  y = cypsel$Adjustment - 100,
  preds = preds,
  beta_scale = 8
)
meds_dat <- addhspars(meds_dat, preds)
meds_dat <- adddisppars(meds_dat)

rhsfit <- stan("rhs_noicpt_wght.stan", data = meds_dat, seed = 142)
plot_mikado(rhsfit)
```

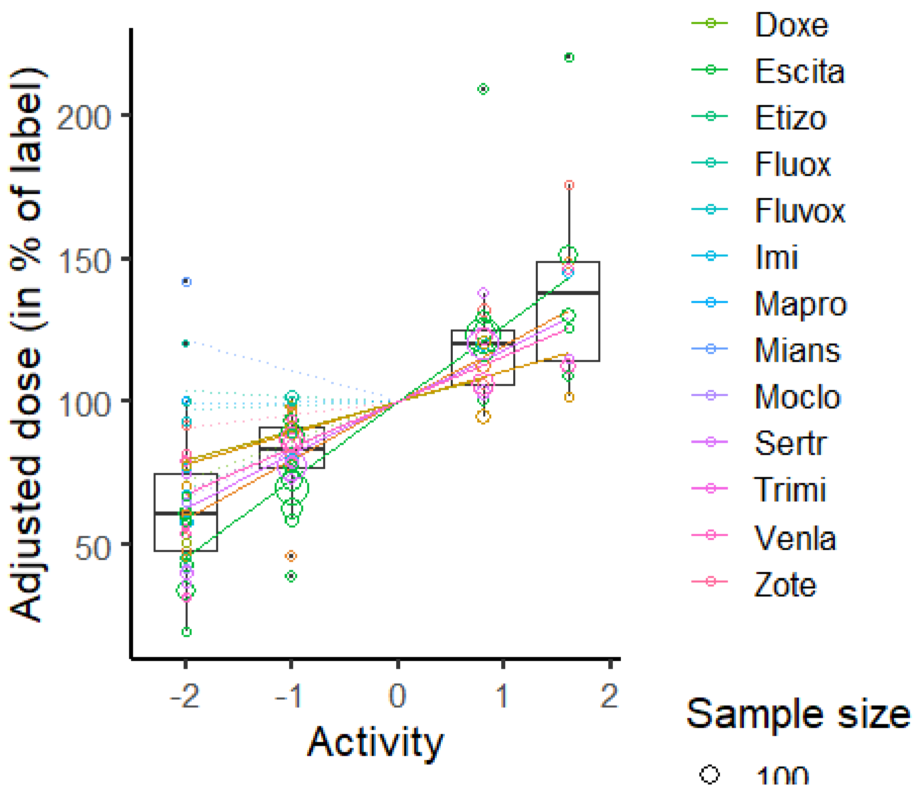


```
plot_intervals(rhsfit, "IM")
```

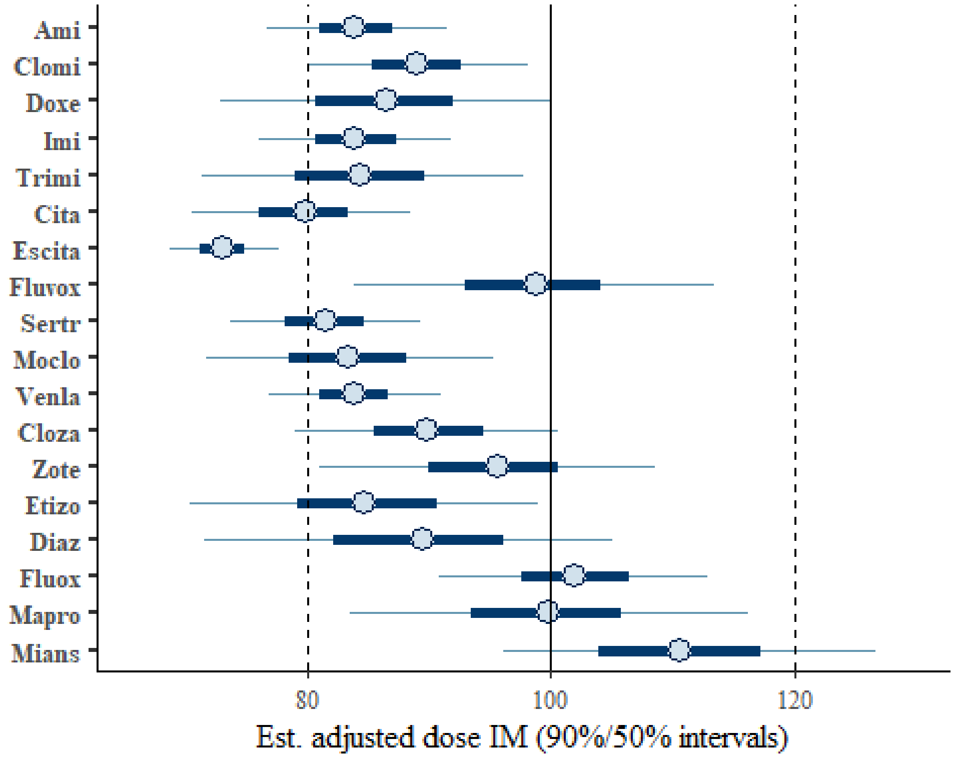

According to this plot, for several substances there is evidence of a CYP2C19 metabolic pathway, but the estimation the effect of the polymorphism is based on so little data that an adjusted dose cannot be formulated precisely. There is good evidence for an adjustment of 80% of the dose label in citalopram, sertraline, venlafaxine, and of 70% in escitalopram.

Below, we display the estimated fit for each substance (in blue), together with the data from the studies and the resulting boxplots; for comparison, we display adjustments computed with the traditional method (separate averages in each substance-phenotype group) in orange. One can see that substances from studies with small samples have coefficients that are much smaller than in the study data. In comparison to the adjustments with the traditional method, they are much more conservative except when there are many data from which the adjustment may be estimated.

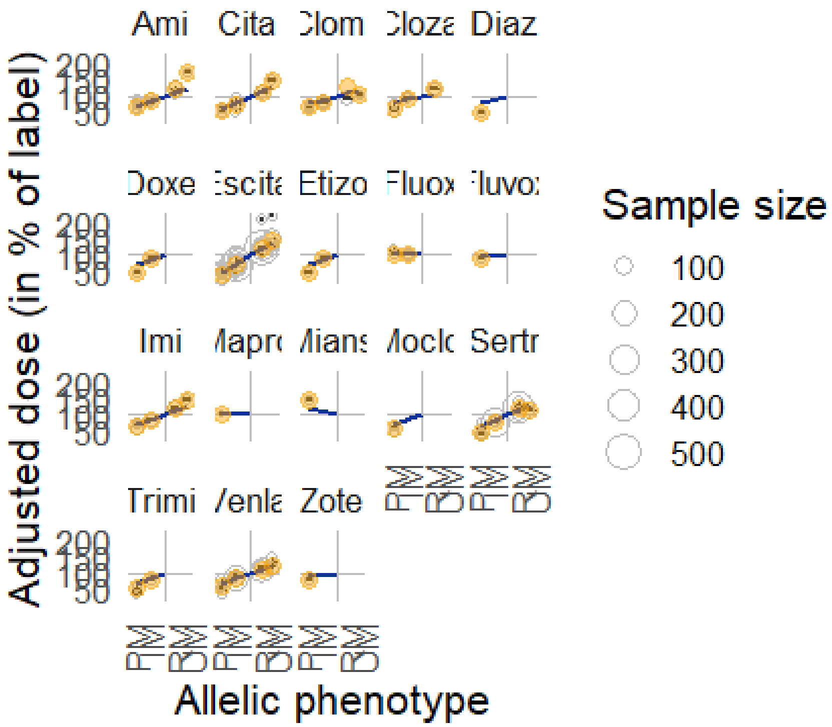

As in the previous model, stan diagnostic were fine here. Because this is the main model of the study, we show samples for the posterior for key variables.

```
df <- as.data.frame(rhsfit)
hist(log(df$tau))
```

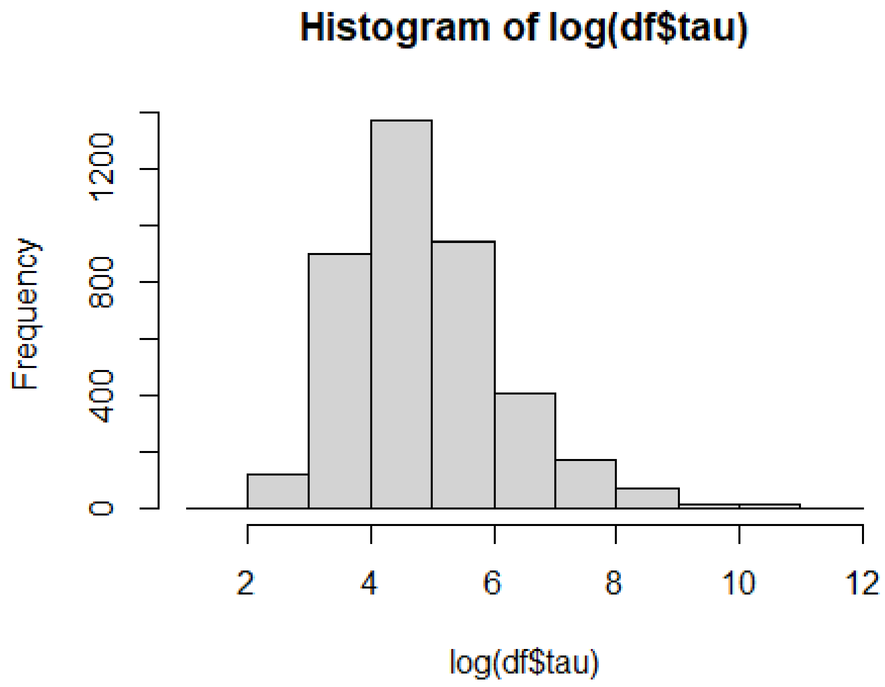

```
hist(log(df$c))
```

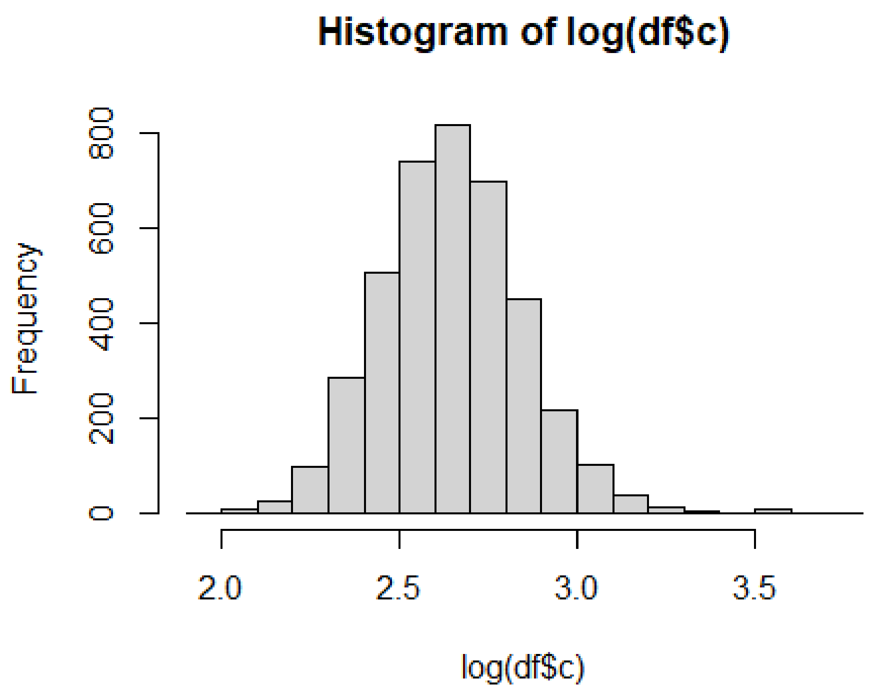


```
hist(df$sigma_within)
```

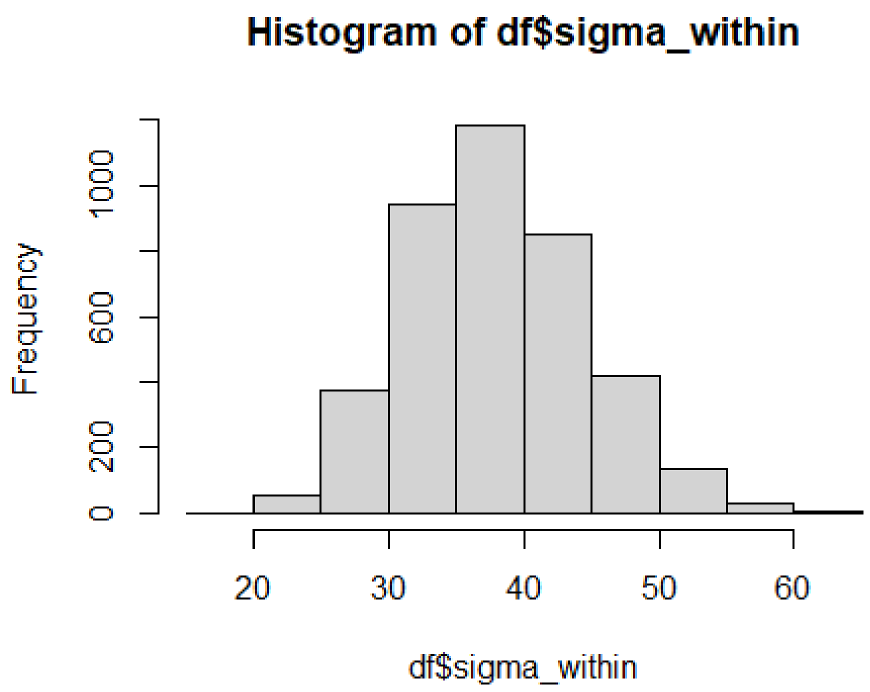


```
hist(df$sigma_betw)
```

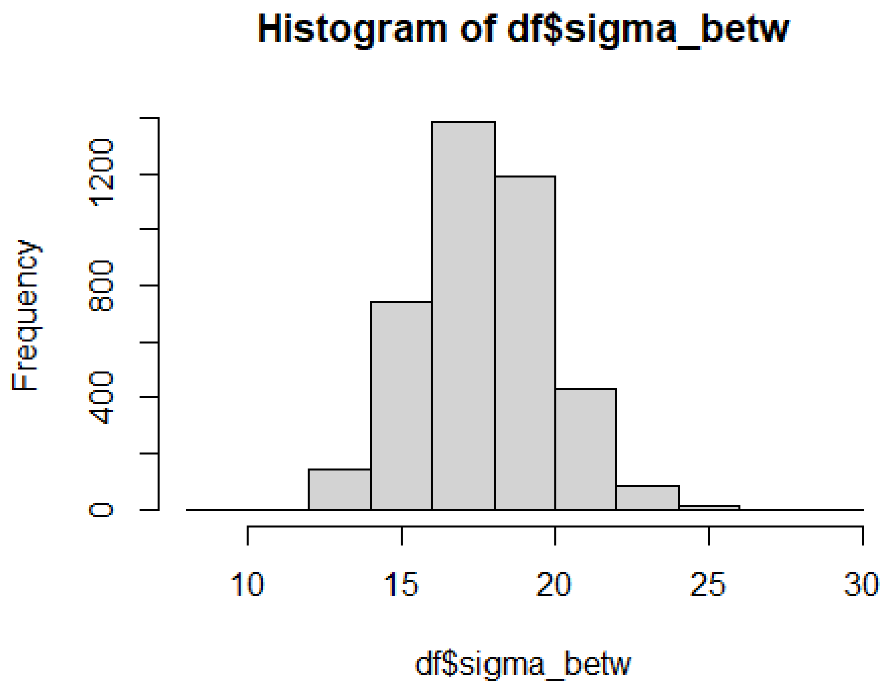


```
mcmc_intervals(rhsfit, regex_pars = "^sigma")
```

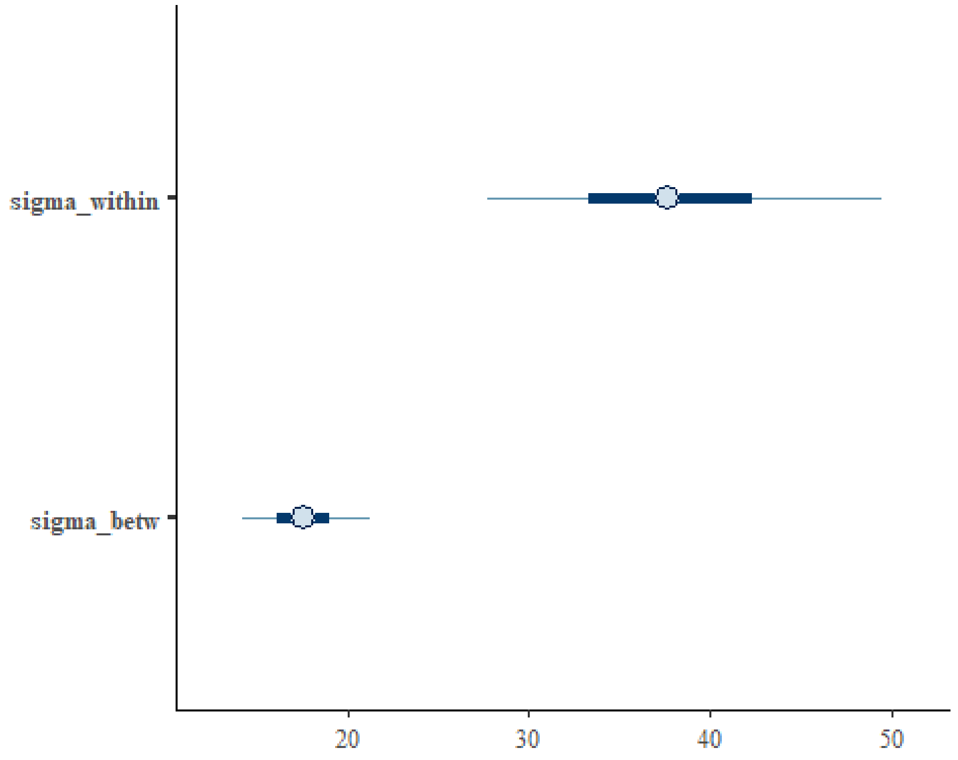


```
mcmc_intervals(rhsfit, regex_pars = "lambda\\[", transformations = log)
```

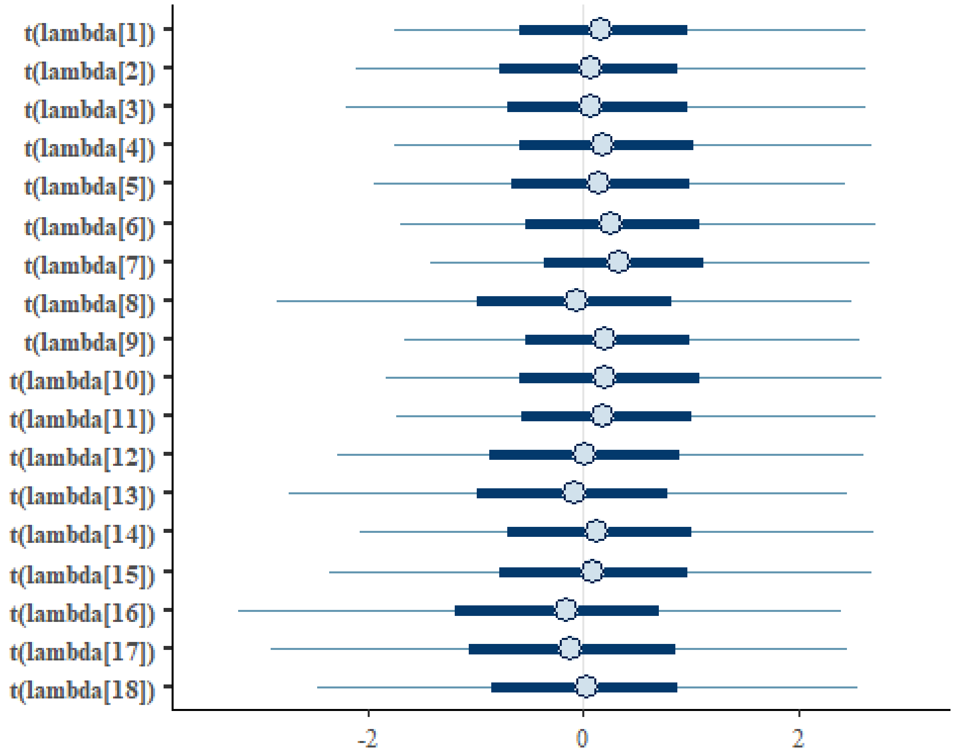


```
mcmc_intervals(rhsfit, regex_pars = "lambda_tilde", transformations = log)
```

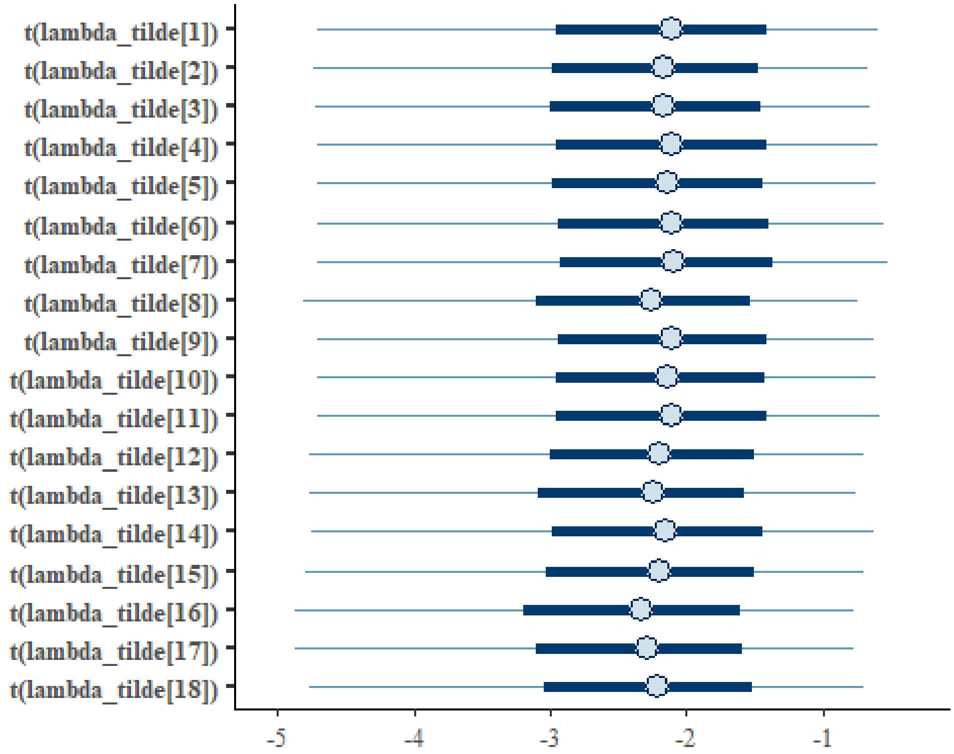


```
rm(df)
```

The distribution of the fitted $\lambda_{j[i]}$ suggests that the regularized horseshoe is penalizing all parameters equally. Note also the sampling of sigma2 suggests the existence of variability between studies that is not quenched by increasing sample size, in effect setting a bound to precision attainable to large samples. The effect of modelling residuals as the sum of two components, within- and between-datapoints variance, is to moderate the contribution of very large studies in the estimation of credibility intervals, which are wider than they would be when modelling residuals by weighting directly by sample size.

The residuals are fairly symmetrical around zero, indicating no need to take logs of adjustments:

```
plot_mikado_res(rhsfit)
```

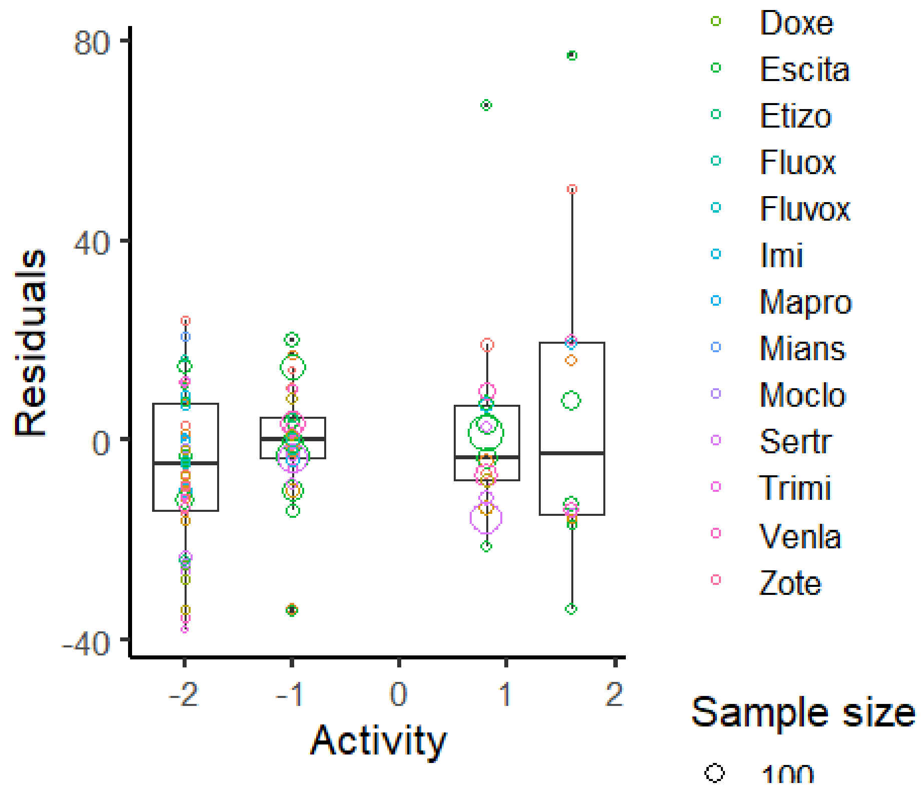

Horseshoe model, without regularization

This model replaces the horsehoe prior for the random effect activity scores x substances with a ridge without regularization (see text for details; the model is in the hs_noicpt_wght.stan file).

```
meds_dat$scale_local <- 1   #Cauchy+(0, 1)
hsfit <- stan("hs_noicpt_wght.stan", data = meds_dat, seed = 142,
              control = list(adapt_delta=0.99, stepsize=0.001))

## Warning: There were 31 divergent transitions after warmup. See
## https://mc-stan.org/misc/warnings.html#divergent-transitions-after-warmup
## to find out why this is a problem and how to eliminate them.
```

```
## Warning: There were 12 transitions after warmup that exceeded the maximum
treedepth. Increase max_treedepth above 10. See
## https://mc-stan.org/misc/warnings.html#maximum-treedepth-exceeded

## Warning: Examine the pairs() plot to diagnose sampling problems
```

```
plot_mikado(hsfit)
```

```
## `summarise()` has grouped output by 'Substance'. You can override using
the
## `.groups` argument.
```

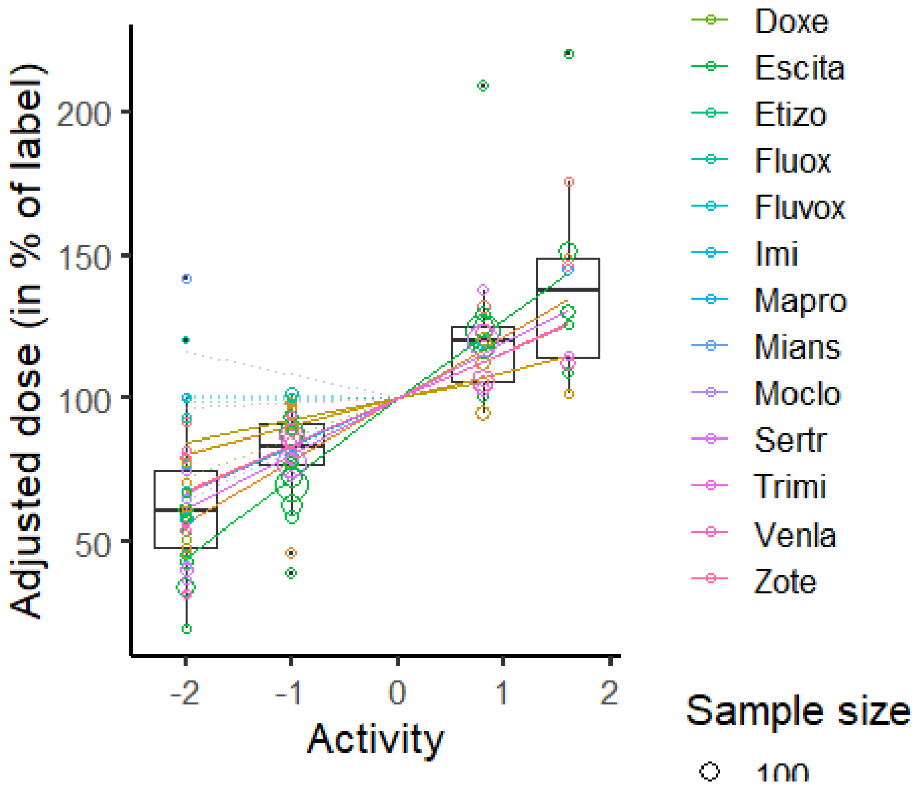


```
plot_intervals(hsfit, "IM")
```

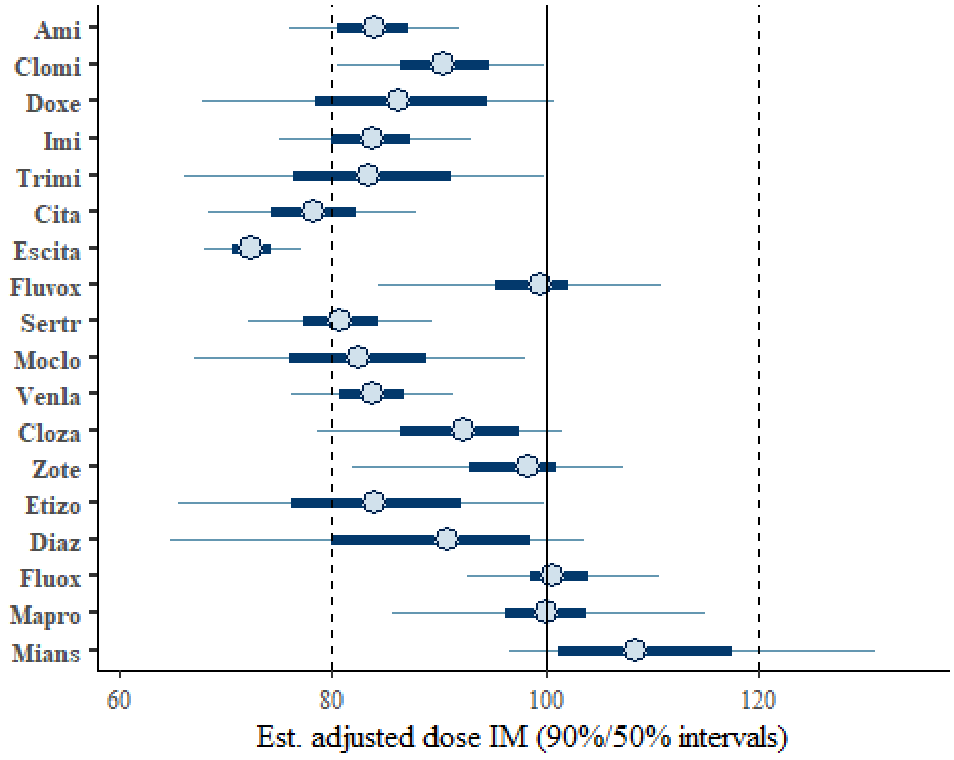

The outcome of this model is similar to the previous model, but there were divergent transitions when sampling from the posterior that we could not eliminate. However, since the results were similar with thouse of the regularized model, we considered the fit as acceptable.

```
df <- as.data.frame(hsfit)
hist(log(df$tau))
```

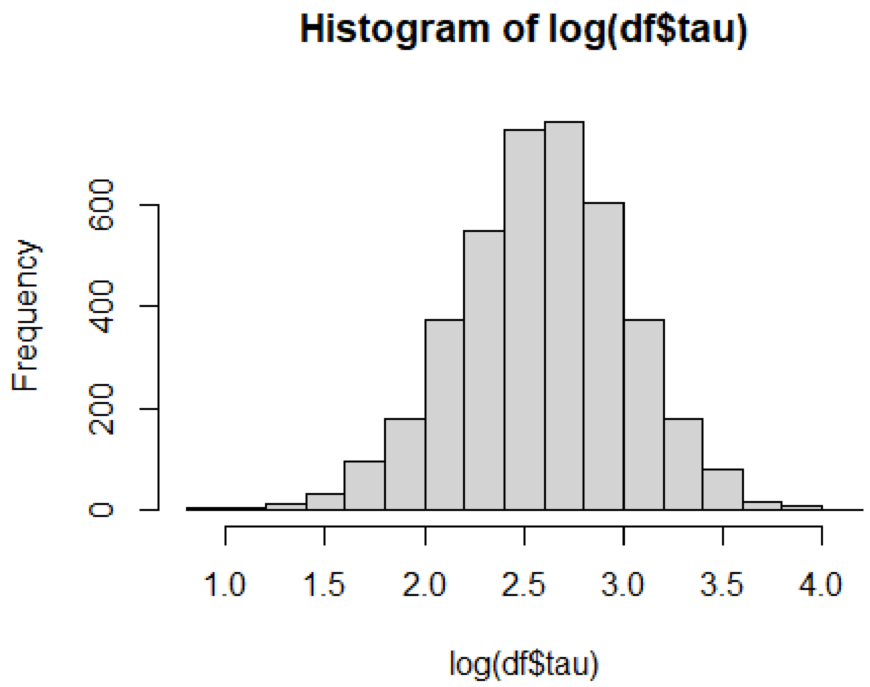


```
hist(df$sigma_within)
```

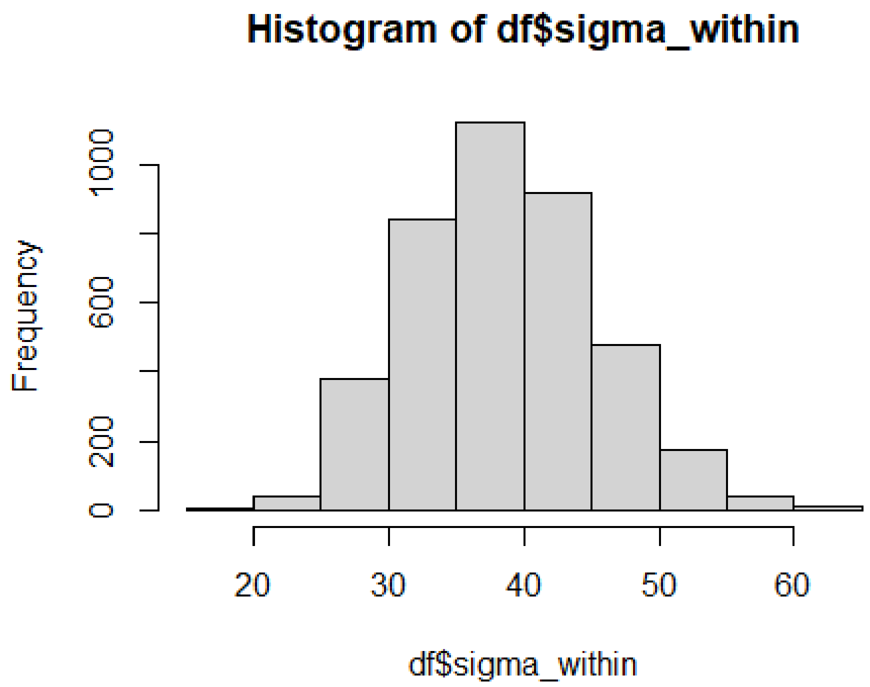


```
hist(df$sigma_betw)
```

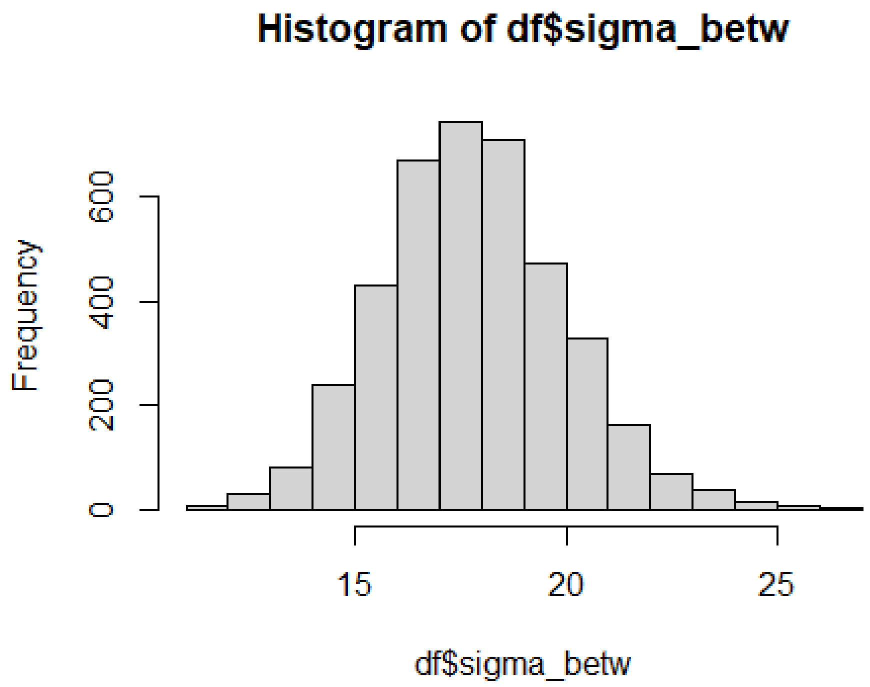


```
mcmc_intervals(hsfit, regex_pars = "^sigma")
```

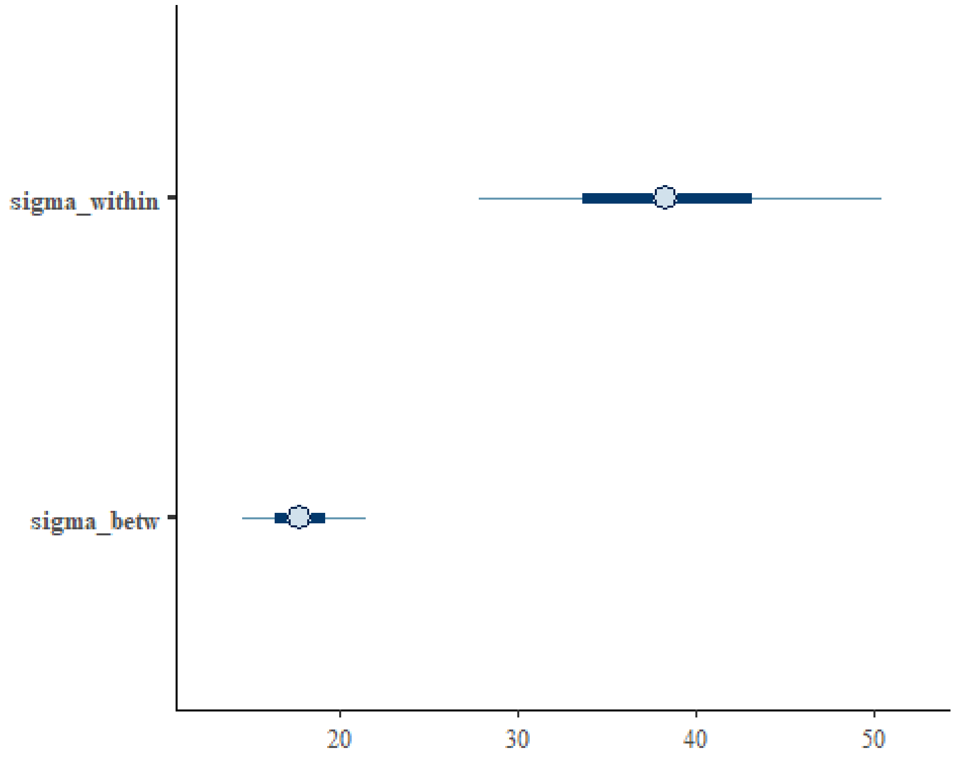


```
mcmc_intervals(hsfit, regex_pars = "lambda\\[", transformations = log)
```

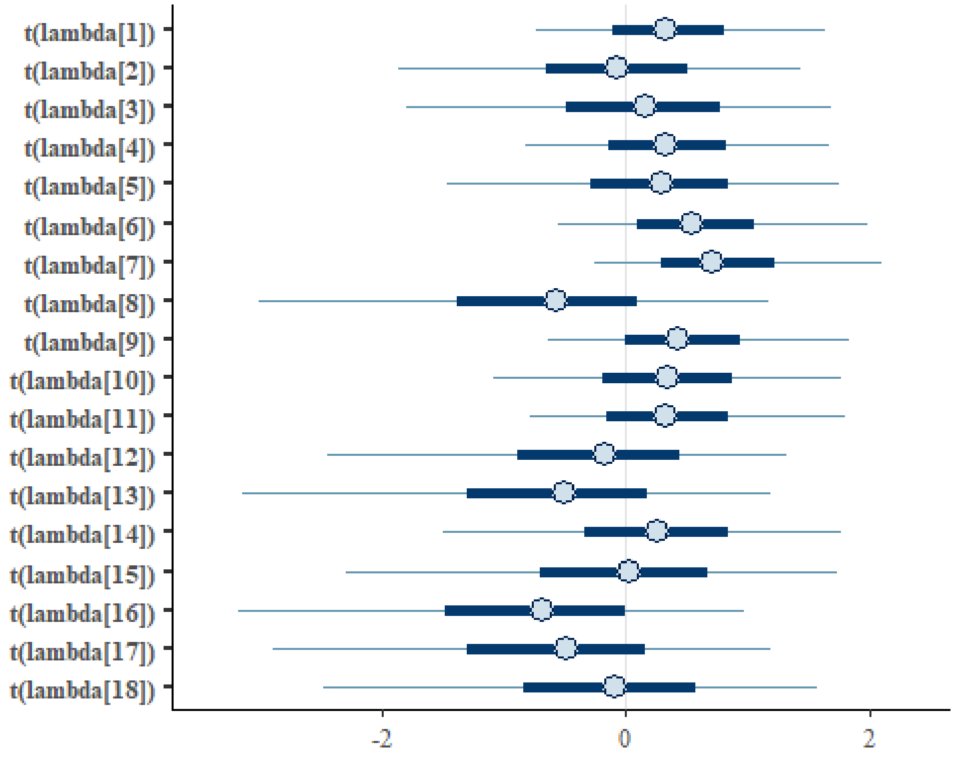


```
rm(df)
```

"Fixed" effects

The model for the fixed effects replaces the horseshoe prior with separate flat priors for the interactions of activity scores with substances. The model for weighting for sample size is the same as in the horseshoe model (the model is in the fixed_noipct_wght.stan file)

```
meds_dat$preds <- cypsel %>%
  pivot_wider(names_from = Substance, values_from = Activity,
              values_fill = 0) %>%
  select(Ami:Mians, RM_1717) %>% as.matrix()
meds_dat$M <- ncol(meds_dat$preds)

fixfit <- stan("fixed_noipct_wght.stan", data = meds_dat, seed = 142)
plot_mikado(fixfit)

## `summarise()` has grouped output by 'Substance'. You can override using
the
## `.groups` argument.
```

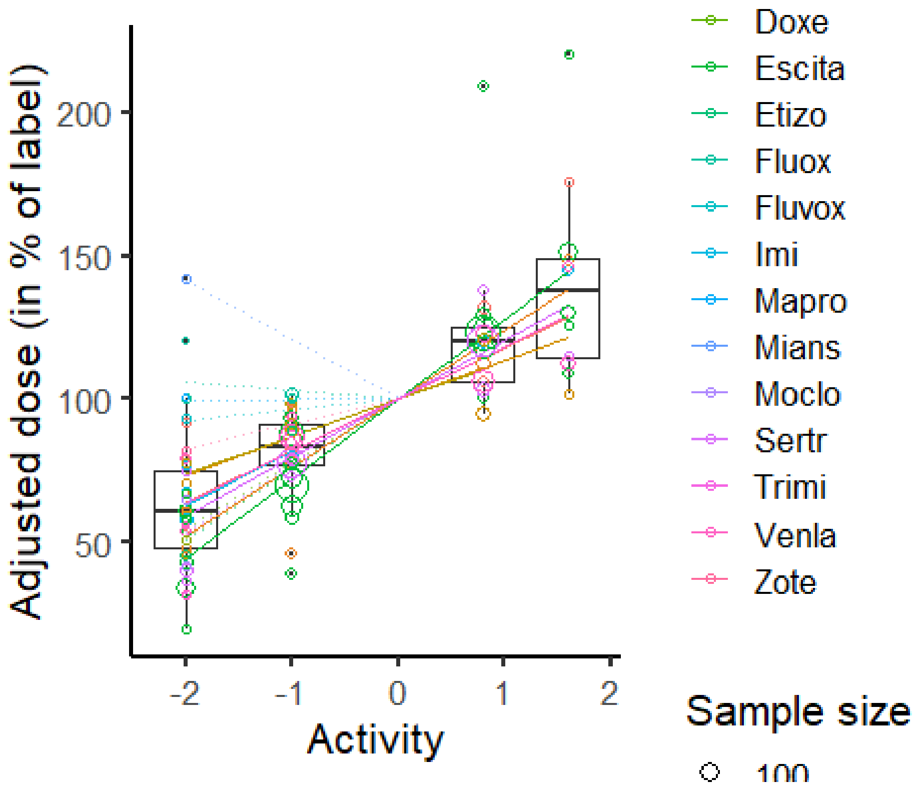


```
plot_intervals(fixfit, "IM") #+ scale_x_continuous(limits = c(40, 140))
```

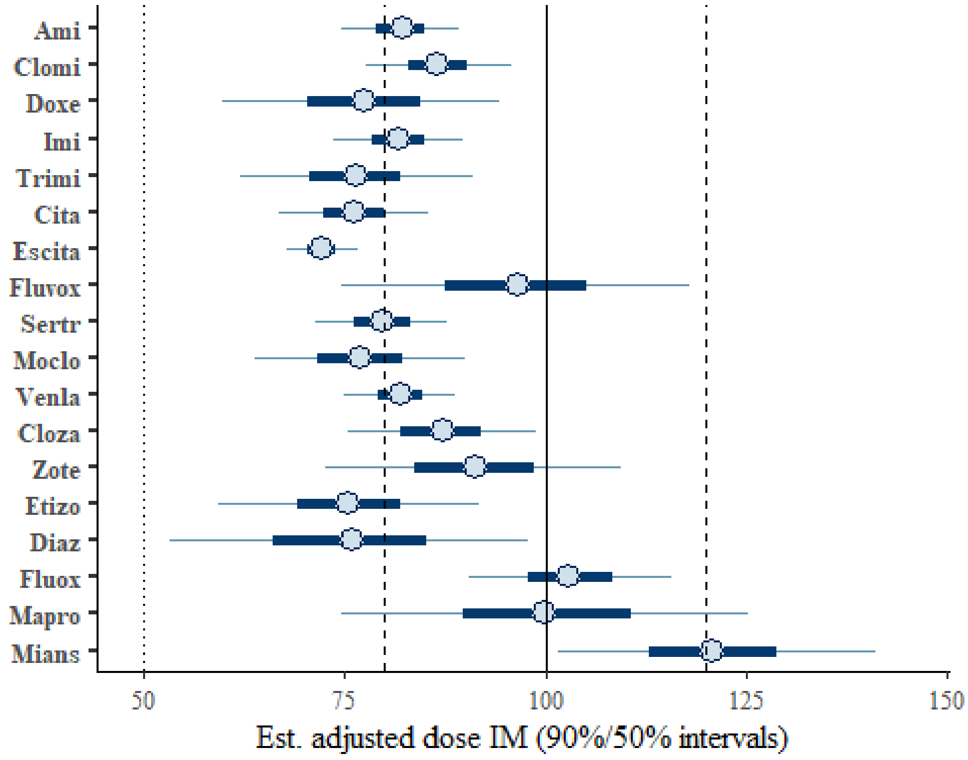

This model essentially fits the groups separately (no shrinkage), so it follows the data closely. The plot of the estimated fit for each substance (in blue), together with the data

from the studies and the resulting boxplots (below) shows the adjustments following the data irrespective of data size. The adjustments computed with the traditional method (separate averages in each substance-phenotype group) in orange follow the original data even more closely. This is due to the fact that here the adjustments are computed from the estimated strength of the metabolic pathway, so even the fixed effects approach leads to more consistent estimates from pooling all data about one substance.

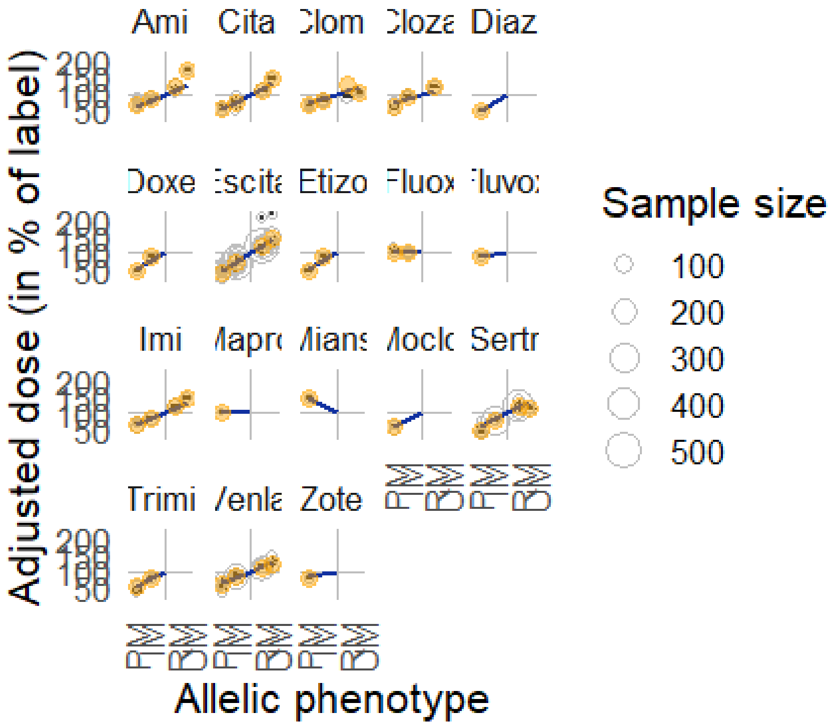

Plotting models together

We plot adjustments for all these models together to compare their performance.

```
plot_multiple_intervals(c(fixfit, rndfit, hsfit, rhsfit),
                        c("fixed", "gauss", "horseshoe", "reghorsesh"), "IM")
```

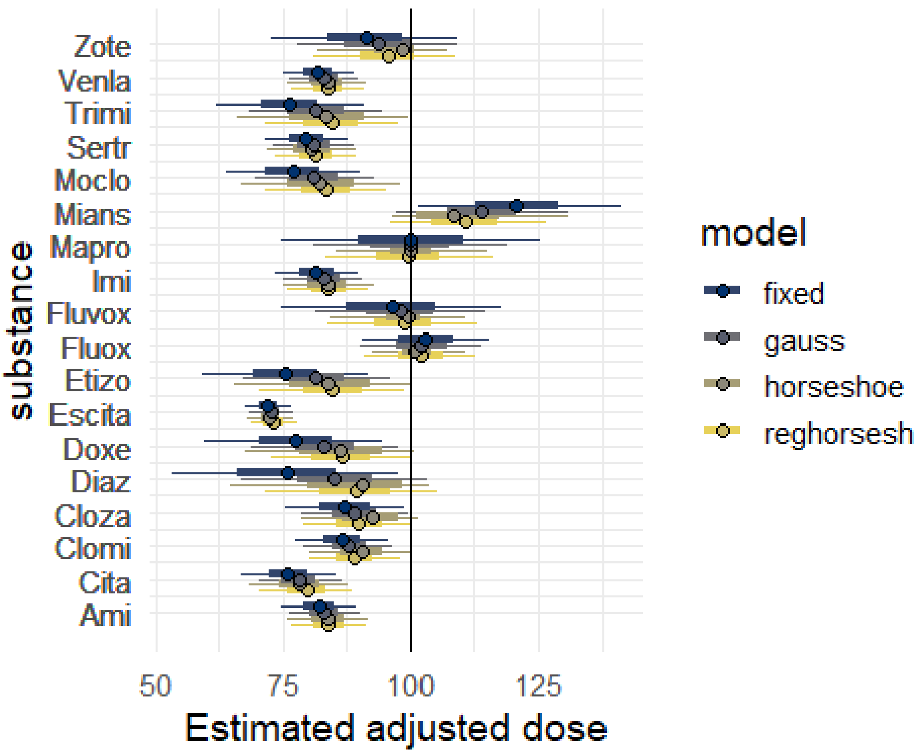

One can see here that the Gaussian model leads to intermediate shrinkage values between the fixed effects model and the horseshoe. The regularized and the non-regularized horseshoe give similar results, but the former is much easier to fit and we therefore prefer it.

Sensitivity analysis

We conducted a sensitivity analysis for the model we considered the best to model dose adjustment, i.e. the regularized horseshoe (with pooled *17 homzygotes in the RM group as confounder).

The most important parameter is the prior for τ. Based on a prior setting 50% effects away from zero, we set in the main analysis the prior for τ to $\tau \sim C^{+}(0,0.1)$. We now conduct a sensitivity analysis where we set this prior to a much more prudential value, $\tau \sim C^{+}(0,0.001)$.

```
# change only global scale for tau
meds_dat$scale_global <- 0.001
rhsfit_001 <- stan("rhs_noicpt_wght.stan", data = meds_dat, seed = 142)
plot_mikado(rhsfit_001)
```

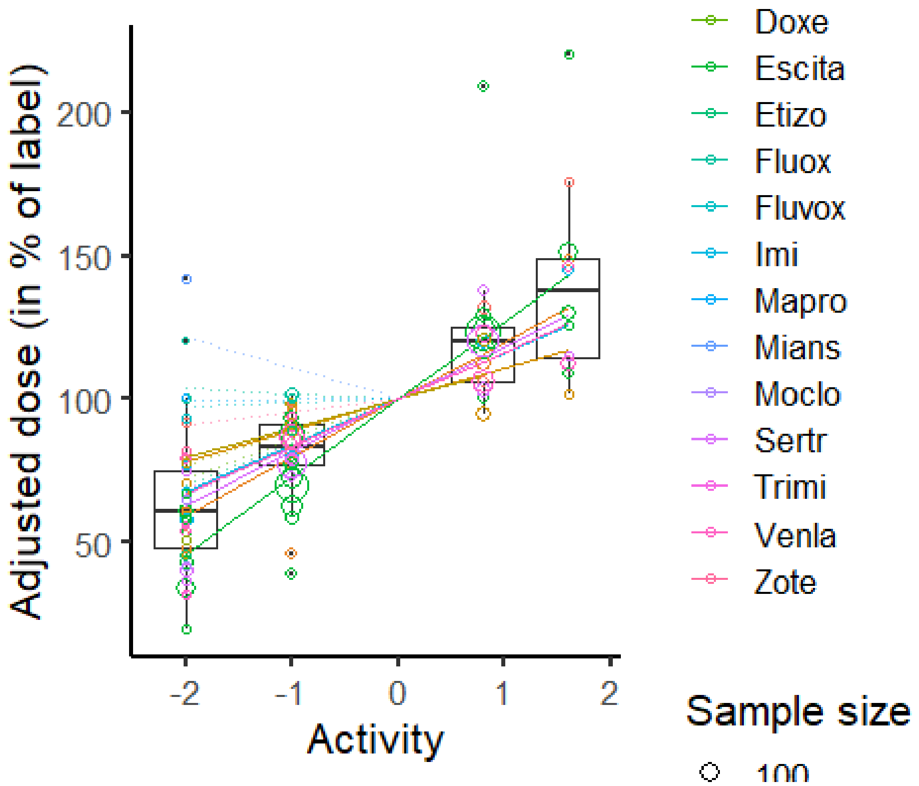


```
plot_intervals(rhsfit_001, "IM")
```

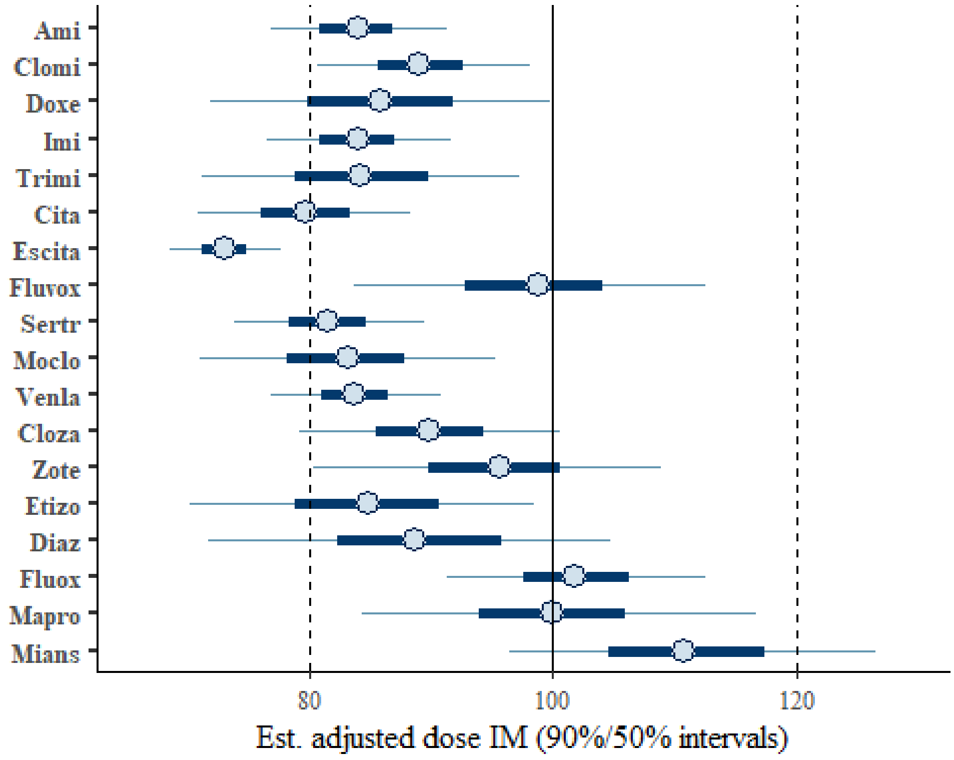

One can see that the outcome is very similar to the original model, showing that the data identify τ:

```
df <- as.data.frame(rhsfit_001)
hist(log(df$tau))
```

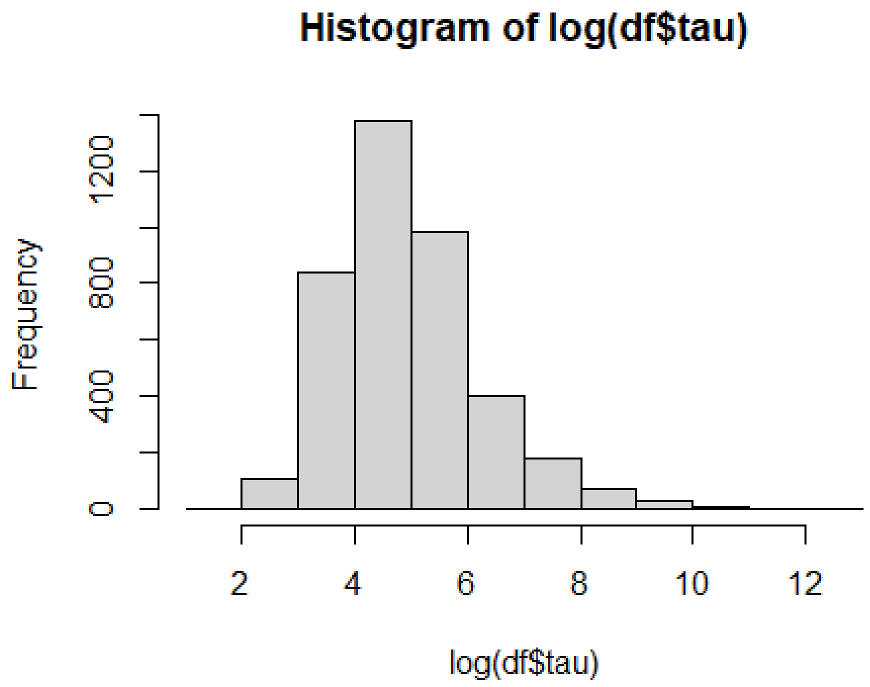

As a result, estimates of dose adjustments are not affected by this prior setting:

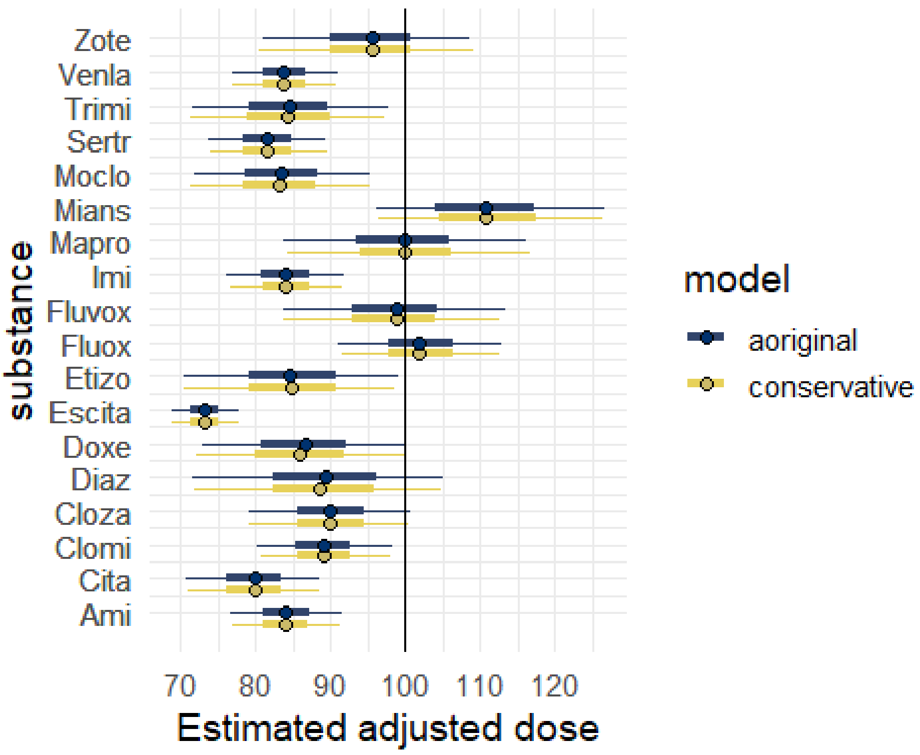

In blue, predicted adjustments of the original model (prior 50% true effects); in yellow, conservative prior

In Supplementary file S2, we showed that study properties (pharmacokinetic method, single/multiple dose studies) were associated with each other, mainly depending on when the study was carried out. Because more recent studies tended to investigate substances with known effects, effects of these properties are confounded by this knowledge and are likely not appropriate confounders. However, we may estimate differences in variability, which may be lower in older models after adjusting for pooled genotypes. For a sensitivity analysis, we only consider single/multiple dose as a proxy for these properties, and model residual errors as follows, replacing variance within σ_w^2 with σ_k^2,

$$\epsilon_i \sim N(0, \sigma_k^2/n_i + \sigma_b^2), k = 1,2$$

where k indexes single and multiple dose, and i indexes the datapoints as before, $i = 1,2, \dots, N$. We keep the adjustment for RM pooling in the model (in file rhs_noicpt_wghtex.stan).

```
#named list for stan. Confounding covariates at the end.
preds <- cypsel %>%
  pivot_wider(names_from = Substance, values_from = Activity,
              values_fill = 0) %>%
  mutate(Activity = get_activity(Phenotype)) %>%
  dplyr::select(Ami:Mians, RM_1717) %>% as.matrix()
mednames <- colnames(cypsel %>%
                       pivot_wider(names_from = Substance, values_from =
Activity,
                                   values_fill = 0) %>%
```

```
                        dplyr::select(Ami:Mians))

meds_dat <- list(
  N = nrow(preds),
  K = 18,
  M = ncol(preds),
  nobs = cypsel$Size,
  #MD modelled at index 1, SD at index 2
  sigmaidx = as.integer(cypsel$Dosage == "SD") + 1,

  y = cypsel$Adjustment - 100,
  preds = preds,
  beta_scale = 8
)
meds_dat <- addhspars(meds_dat, preds)
meds_dat <- adddisppars(meds_dat)

rhsfitex <- stan("rhs_noicpt_wghtex.stan", data = meds_dat, seed = 142)
print(rhsfitex, par = c("sigma_within", "sigma_betw"))

## Inference for Stan model: anon_model.
## 4 chains, each with iter=2000; warmup=1000; thin=1;
## post-warmup draws per chain=1000, total post-warmup draws=4000.
##
##                  mean se_mean   sd  2.5%   25%   50%   75% 97.5% n_eff
Rhat
## sigma_within[1] 47.73    0.14 8.38 31.98 41.85 47.49 53.31 64.70  3517
1
## sigma_within[2] 37.62    0.13 7.73 24.95 32.03 36.85 42.38 54.57  3359
1
## sigma_betw      16.52    0.04 2.27 12.35 14.93 16.43 17.98 21.37  2685
1
##
## Samples were drawn using NUTS(diag_e) at Tue Nov 22 06:06:35 2022.
## For each parameter, n_eff is a crude measure of effective sample size,
## and Rhat is the potential scale reduction factor on split chains (at
## convergence, Rhat=1).
```

As expected, the variance of studies with single dose (sigma_within[2]) is lower than in multiple dose studies (sigma_within[1]). The plot for the estimated variance for different sample sizes is similar to the one of the model in Supplementary File S2:

```
disp <- as.data.frame(rhsfitex) %>% select(matches("^disp")) %>%
map_dbl(median)
plot(disp + runif(nrow(cypsel)) ~ log(cypsel$Size), pch = "o", col =
ifelse(cypsel$Dosage == "SD", "blue", "red"), xlab = "log sample size", ylab
= "dispersion")
```

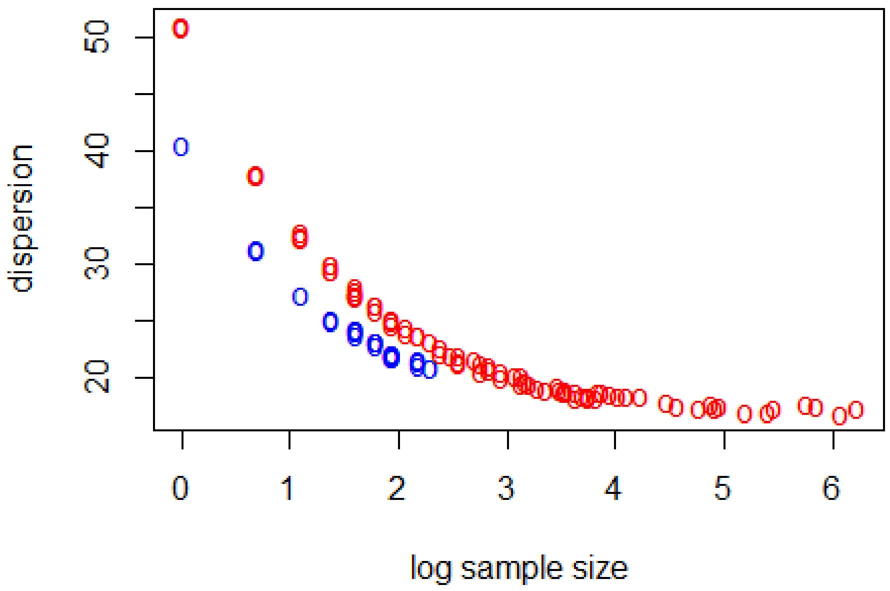

In blue, estimated within datapoint standard deviation of single dose studies, in red, the multiple dose studies, plotted as a function of datapoint sample size. Jitter was added to identify samples.

However, when we compare estimated dose adjustments, there are hardly differences:

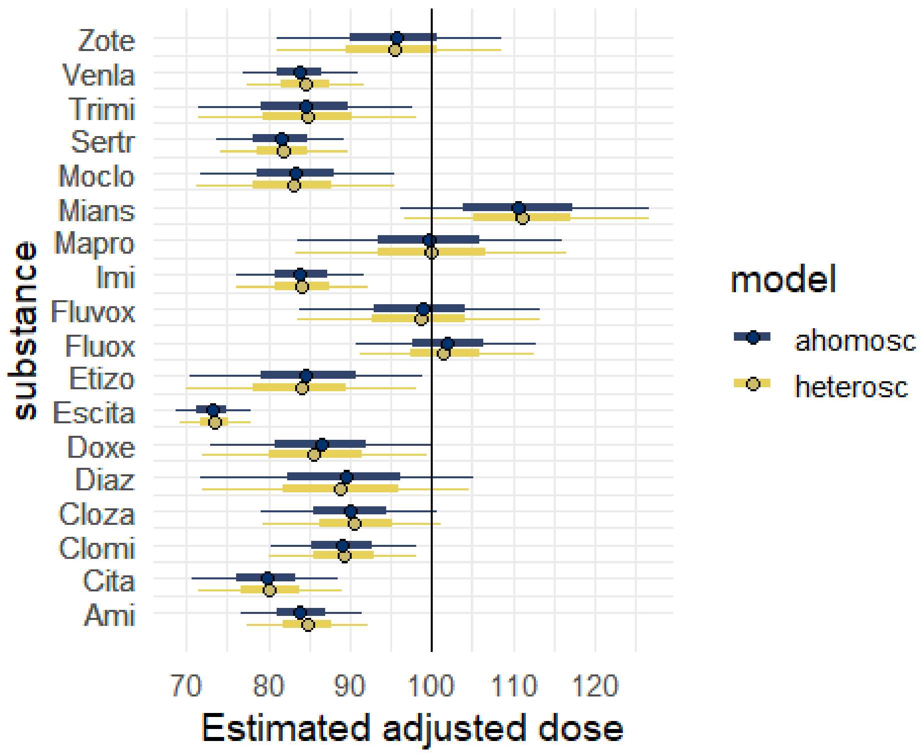

In blue, predicted adjustments of the original model; in yellow, heteroscedastic model (single/multiple dose)

This may be due to the fact that single studies are fewer and conducted with few subjects; the large variance-within of multiple dose studies is compensated by the large number of participants.

We conclude the model to be appropriate without modelling heteroscedasticity.
