# Supplementary material for: Pharmacogenetic Dose Modeling Based on CYP2C19 Allelic Phenotypes
Source: Pharmaceutics. 2022 Dec 16;14(12):2833. doi: 10.3390/pharmaceutics14122833 (PMC9781550; doi:10.3390/pharmaceutics14122833)
Supplement: Supplementary file 1 [file pharmaceutics-14-02833-s001.zip › SupplFile S4.zip!/Project/Depends.html]

Dependencies


# Dependencies

#### Roberto Viviani, University of Innsbruck

#### 2022-11-17

## Session information

```
sessionInfo()
```

```
## R version 4.2.0 (2022-04-22 ucrt)
## Platform: x86_64-w64-mingw32/x64 (64-bit)
## Running under: Windows 10 x64 (build 19044)
## 
## Matrix products: default
## 
## locale:
## [1] LC_COLLATE=English_United Kingdom.utf8 
## [2] LC_CTYPE=English_United Kingdom.utf8   
## [3] LC_MONETARY=English_United Kingdom.utf8
## [4] LC_NUMERIC=C                           
## [5] LC_TIME=English_United Kingdom.utf8    
## 
## attached base packages:
## [1] stats     graphics  grDevices utils     datasets  methods   base     
## 
## other attached packages:
##  [1] bayesplot_1.9.0     rstan_2.26.13       StanHeaders_2.26.13
##  [4] lme4_1.1-29         Matrix_1.4-1        purrr_0.3.4        
##  [7] stringi_1.7.8       ggplot2_3.3.6       dplyr_1.0.10       
## [10] tidyr_1.2.0        
## 
## loaded via a namespace (and not attached):
##  [1] tidyselect_1.2.0   xfun_0.31          bslib_0.4.0        splines_4.2.0     
##  [5] lattice_0.20-45    V8_4.2.1           colorspace_2.0-3   vctrs_0.4.2       
##  [9] generics_0.1.3     htmltools_0.5.2    stats4_4.2.0       loo_2.5.1         
## [13] yaml_2.3.6         utf8_1.2.2         rlang_1.0.6        pkgbuild_1.3.1    
## [17] jquerylib_0.1.4    pillar_1.8.1       nloptr_2.0.3       glue_1.6.2        
## [21] withr_2.5.0        matrixStats_0.62.0 lifecycle_1.0.3    stringr_1.4.1     
## [25] munsell_0.5.0      gtable_0.3.1       codetools_0.2-18   evaluate_0.15     
## [29] inline_0.3.19      knitr_1.39         callr_3.7.2        fastmap_1.1.0     
## [33] ps_1.7.0           parallel_4.2.0     curl_4.3.2         fansi_1.0.3       
## [37] Rcpp_1.0.9         scales_1.2.1       cachem_1.0.6       RcppParallel_5.1.5
## [41] jsonlite_1.8.2     gridExtra_2.3      digest_0.6.29      processx_3.7.0    
## [45] grid_4.2.0         cli_3.3.0          tools_4.2.0        magrittr_2.0.3    
## [49] sass_0.4.2         tibble_3.1.8       crayon_1.5.2       pkgconfig_2.0.3   
## [53] ellipsis_0.3.2     MASS_7.3-56        prettyunits_1.1.1  ggridges_0.5.4    
## [57] minqa_1.2.4        rmarkdown_2.17     rstudioapi_0.13    R6_2.5.1          
## [61] boot_1.3-28        nlme_3.1-157       compiler_4.2.0
```
